# Developing a Morphing Taxidermy of a Red-Tailed Hawk

**DOI:** 10.3390/biomimetics11080559

**Published:** 2026-08-05

**Authors:** Peter L. Bishay, Estefany Ortega, Leo Haroutoonian, Victoria Bures, Johnathon Moore, James Hogue, Fritz Hertel

**Affiliations:** 1 Department of Mechanical Engineering, California State University, Northridge, CA 91330, USA; 2 Department of Biology, California State University, Northridge, CA 91330, USA; james.n.hogue@csun.edu (J.H.); fritz.hertel@csun.edu (F.H.)

**Keywords:** biomimetic, wing-morphing, taxidermy, bioinspiration, avian flight

## Abstract

Studying the flight of birds can provide a lot of inspiration for the design of future drones and airplanes and deepen our understanding of bird ecology. Fixed taxidermies have been used in ecological studies and aerodynamic investigations of avian flight. Building morphing bird taxidermies has the potential to extend future studies incorporating aspects of bird flight while avoiding the challenges of live bird testing. However, creating such morphing taxidermies imposes serious challenges, such as the limited space inside the bird’s dead body for electronic components and the rapid desiccation of the integument. This work presents a first successful attempt to build a morphing taxidermy of a red-tailed hawk. Novel mechanisms were developed to morph the wings and tail of the prototype, which includes real feathers and is covered by the bird’s real skin. The completed morphing taxidermy features wings that morph independently, a tail with pitch, roll, and feather-spread capabilities, along with a fuselage that interconnects all systems with the bird’s carcass. Actuation tests demonstrated the effectiveness of the proposed design and validated the design choices. Multi-cycle actuation tests on the proof-of-concept model demonstrated morphing consistency. The paper is method-focused, and application studies are forthcoming.

## 1. Introduction

Birds represent one of the most sophisticated examples of natural flight systems, combining lightweight skeletal structures, flexible feathers, and highly adaptable wing and tail morphologies. Unlike conventional fixed-wing aircraft, birds continuously modify wing sweep, span, camber, twist, and tail geometry in response to changing aerodynamic and ecological demands. These wing and tail morphing capabilities enable birds to adapt to wind changes, snatch prey using their claws, and make sharp turning maneuvers. These morphing capabilities contribute to flight efficiency, maneuverability, stability, and control, making birds an important source of inspiration for both biological research and aerospace engineering [1,2].

Numerous studies have investigated the aerodynamic characteristics of bird wings using live birds [1], prepared specimens [3], computational models, and physical replicas. Recent research has demonstrated that birds actively alter wing geometry to regulate aerodynamic efficiency, stability, maneuverability, and control authority [4]. Harvey et al. [5,6] showed that birds can modulate static pitch stability through wing morphing during gliding flight, allowing transitions between stable and unstable flight states depending on flight requirements. Harvey et al. [7] also demonstrated that gull-inspired joint-driven wing morphing can provide adaptive longitudinal flight control, highlighting how biological morphing mechanisms may replace conventional aircraft control strategies.

Several research platforms have attempted to replicate avian morphing mechanisms in engineered flight systems, such as LisHawk [8], LisEagle [9], MataGull [10,11], and CGull [12,13]. Among the most biologically realistic morphing UAVs developed to date are PigeonBot [14] and PigeonBot II [15]. Unlike conventional robotic aircraft, PigeonBot incorporated real pigeon feathers mounted on an articulated skeletal structure to reproduce the aerodynamic behavior of bird wings. The only drone that utilized a bird’s natural parts was the flapping-wing Ornithopter [16]. This drone had a pheasant’s body and pigeon’s wings that could only flap without morphing. Table A1 in Appendix A presents a comparison between some of these platforms and the proposed morphing taxidermy model.

Taxidermy specimens have long been used as research tools in ecological and behavioral studies because they provide standardized and repeatable representations of birds while eliminating behavioral variability. In behavioral ecology, taxidermy mounts have been employed to investigate predator–prey interactions, social signaling, and species recognition. Nebel et al. [17], for example, used taxidermy black sparrowhawks to simulate predator attacks and examine the escape responses of feral pigeons under varying environmental conditions. Similarly, Falk et al. [18] used taxidermy hummingbird mounts to investigate the role of plumage polymorphism and social harassment in female hummingbirds.

Taxidermy birds have increasingly been utilized in aerodynamic investigations due to the preservation of the natural feather arrangement, wing shape, and body geometry of real birds. They offer advantages over simplified engineering models. Kishimoto et al. [19] conducted wind tunnel experiments on taxidermy black-tailed gulls and black kites to investigate aerodynamic characteristics associated with naturally occurring morphing wing configurations. Subsequent investigations using taxidermy black-tailed gulls, albatrosses, and three-dimensional printed models further demonstrated the usefulness of prepared specimens for measuring lift, drag, and stability characteristics under controlled wind tunnel conditions [20,21].

Despite these advantages, conventional taxidermy specimens remain limited because they are generally fixed in a single posture. Wind tunnel experiments have shown that taxidermy wings may deform under aerodynamic loading due to insufficient structural rigidity, resulting in aerodynamic behavior that differs from both rigid engineering models and living birds [20,21]. Birds continuously adjust wingspan, sweep, twist, and tail orientation to optimize performance under changing flight conditions. For example, peregrine falcons adopt a series of distinct wing configurations during high-speed dives and pullout maneuvers, using wing morphing to enhance lift generation, maneuverability, and flight control [2]. Likewise, studies of albatross wing geometry have shown that passive and active morphing significantly alter aerodynamic performance and lift-to-drag ratio [22]. These observations suggest that realistic investigation of avian flight requires not only accurate morphology but also controlled morphing capability.

To overcome the limitations of fixed taxidermy specimens, many researchers have relied on experiments involving live birds. Live-bird wind tunnel studies provide valuable information regarding natural flight behavior, aerodynamic performance, and wing morphing strategies [1,23,24,25]. However, such studies are often difficult to conduct because they require extensive animal training, specialized facilities, ethical approval, and substantial time investment [26].

A morphing wing and tail taxidermy has the potential to address many of these challenges by combining the realism of biological specimens with the controllability of engineered experimental platforms. Unlike conventional taxidermy, a morphing taxidermy could preserve authentic feather structure and species-specific morphology while allowing researchers to actively modify wing and tail configurations. By combining biological realism with experimental control, such a system has the potential to reduce dependence on live-bird experimentation while enabling new investigations into avian flight mechanics, ecology, and bio-inspired aircraft design. To the best of the authors’ knowledge, there is no existing platform that combines authentic avian morphology with controlled wing and tail morphing capabilities. Hence, the objective of this study is to develop a morphing bird taxidermy capable of reproducing biologically relevant wing and tail configurations. This platform is intended to provide a repeatable and ethically responsible alternative to live-bird experimentation while expanding opportunities for future research in avian ecology, flight mechanics, and bio-inspired engineering. The main hypothesis is that a biomimetic mechanism can be designed to replicate the bird’s wing and tail feather expansion where all required electronics are housed inside a sternum-like structure with utilization of the bird’s natural feathers, skin, and some bones. The rest of the paper is organized as follows: Section 2 (Materials and Methods) presents the approach taken to create the morphing taxidermy. Results are presented in Section 3, followed by a Discussion in Section 4. Summary and Conclusions are presented in Section 5.

## 2. Materials and Methods

### 2.1. Measurements

**Figure 1:**
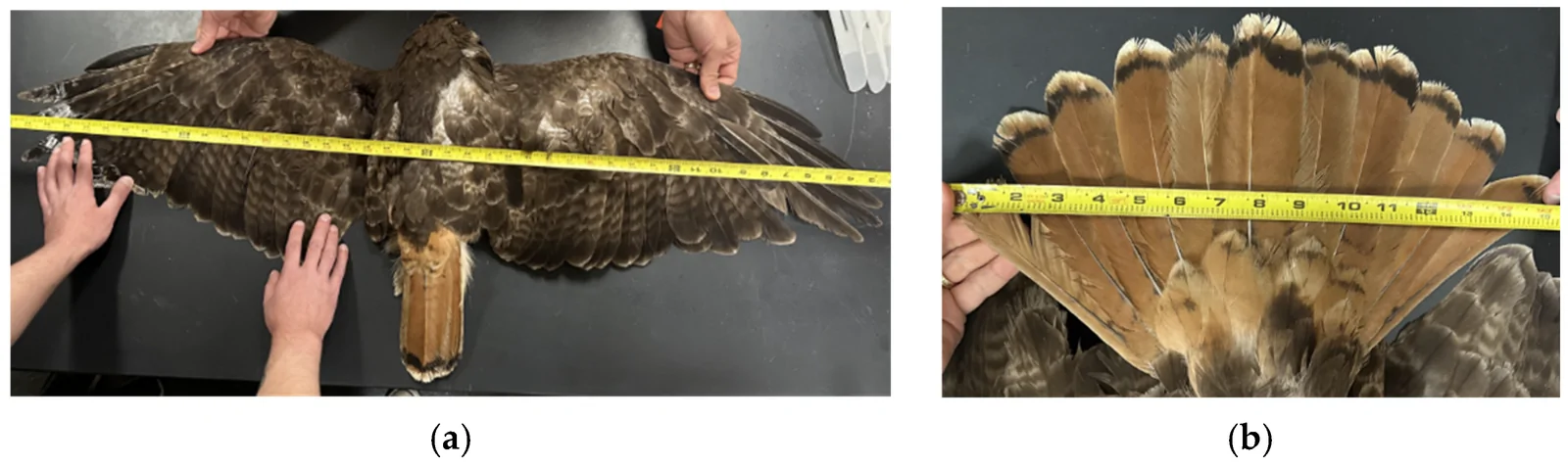
Carcass measurements: (**a**) Wingspan measurement extended configuration; (**b**) Tail measurement extended configuration.

The carcass of a red-tailed hawk (RTH) was obtained from the California Wildlife Center in April 2025. The bird’s exact time of death is unknown, but it was clear from the condition of the carcass that it was placed in a freezer within few hours after its death. Initial measurements of the carcass were taken, as shown in Figure 1a,b, to better understand the bird’s anatomy and to support the design of the mechanical wing and tail mechanisms. Measurements of the approximate wing bone dimensions, wingspan and tail expansion angles were documented and are summarized in Table 1. Overall, the carcass was in good condition for the development of the morphing taxidermy system. Although the bird had sustained an injury to its right wing (belly-up orientation), resulting in a broken humerus bone, most of the wing’s structure and feathers remained intact and undamaged. As a result, the injury did not present a significant limitation to the design and fabrication processes.

### 2.2. Three-Dimensional Scanning

**Figure 2:**
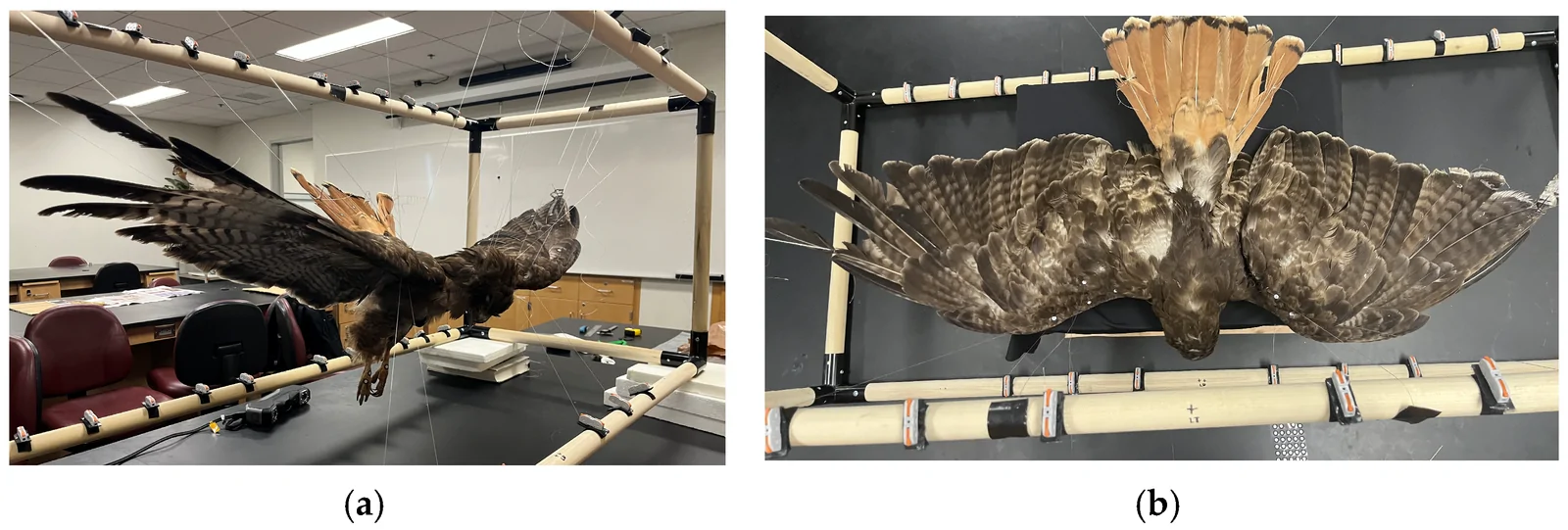
Hawk secured with fishing wire for 3D scan: (**a**) side view; (**b**) top view.

The complete RTH carcass was 3D-scanned using a Revopoint MetroX 3D laser scanner (Revopoint 3D Technologies Inc, Los Angeles, CA, USA). The full-body scan provided dimensional references for the design of the wing and tail assemblies. In addition, the scan was essential for verifying that the designed components would properly fit within the hawk’s body, allowing necessary modifications to be made prior to fabrication. During the scanning process, the hawk was suspended within an open-frame support structure, as shown in Figure 2a,b, using a fishing-line suspension system based on the setup described by Tang et al. [27] for scanning a falcon carcass. The bird was positioned in a fully extended posture, oriented downward and facing forward to enable complete surface capture. To support the torso and prevent sagging, a square piece of stretchable fabric was configured into a hammock-like support beneath the belly using fishing line attached at each corner. Additional fishing lines secured the wings, tail, and body to maintain the extended scanning position throughout the process.

**Figure 3:**
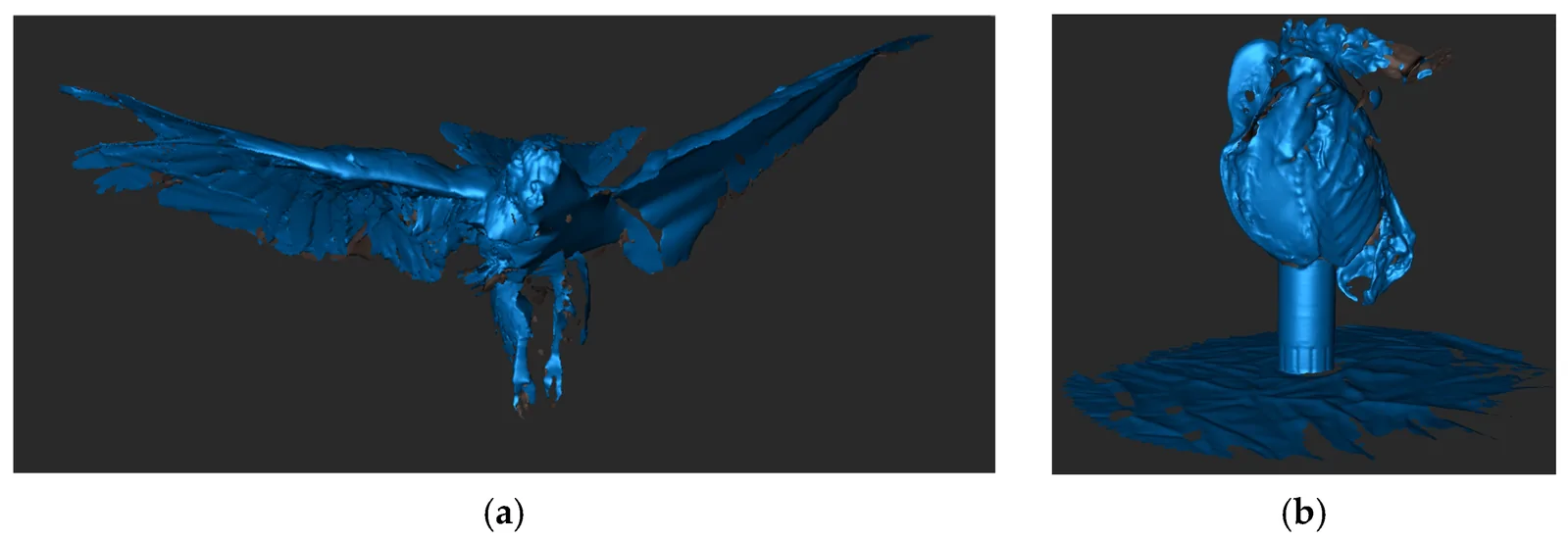
Three-dimensional scan of RTH: (**a**) Front view of the whole model; (**b**) Interior body.

Multiple scans were performed to capture different regions of the cadaver, including the upper surfaces of the wings, the underside of the torso, and the lower surfaces of the wings. These individual scans were then aligned and merged to create a complete 3D model of the hawk, as shown in Figure 3a. To ensure accurate automatic alignment during processing, each scan was captured with approximately 25% overlap relative to adjacent scans. During scanning, the operator manually and slowly moved the scanner around the bird to capture the surface geometry from multiple angles. Additional lighting was also used to improve scan quality and enhance geometric detail capture.

In addition to the full-body scan of the RTH, the torso was separately 3D-scanned following its removal from the hawk (details in Section 2.5), as shown in Figure 3b. This detailed torso scan aided the design of the fuselage (details in Section 2.8). During scanning, the torso was placed on a rotating pedestal while the laser scanner remained stationary, allowing the complete geometry of the torso to be captured as the platform rotated.

**Figure 4:**
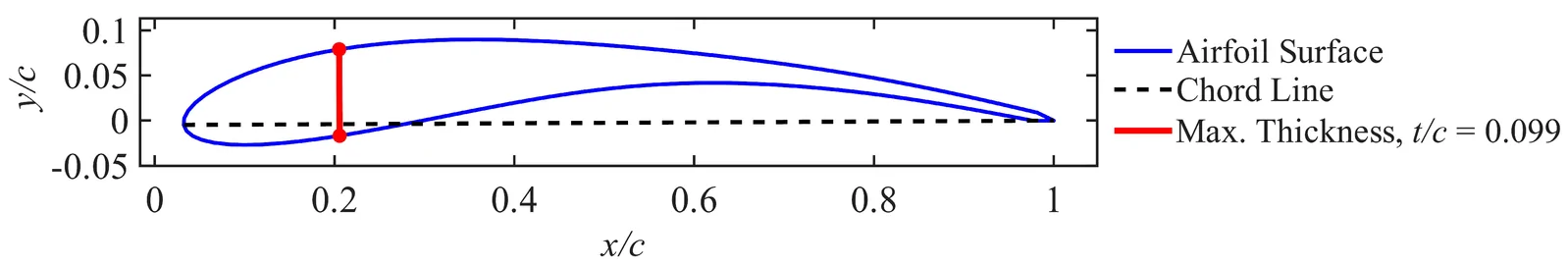
Smoothened RTH Airfoil (*c* is the chord length, *t* is the thickness).

For future mathematical and computational modeling, the airfoil shape of the wing was captured from the 3D scans. The full RTH 3D scan was converted into a SOLIDWORKS (2025) model, where a slice from the mid-wing was taken. The cross-section of this slice was then brought over to Inkscape (1.4.2), wherein the general outline of the wing’s airfoil was obtained using a graph digitization tool. This outline was smoothed using the smoothing spline function on MATLAB (2024) to achieve the RTH airfoil in Figure 4.

### 2.3. Motion Capture of Wings and Tail

**Figure 5:**
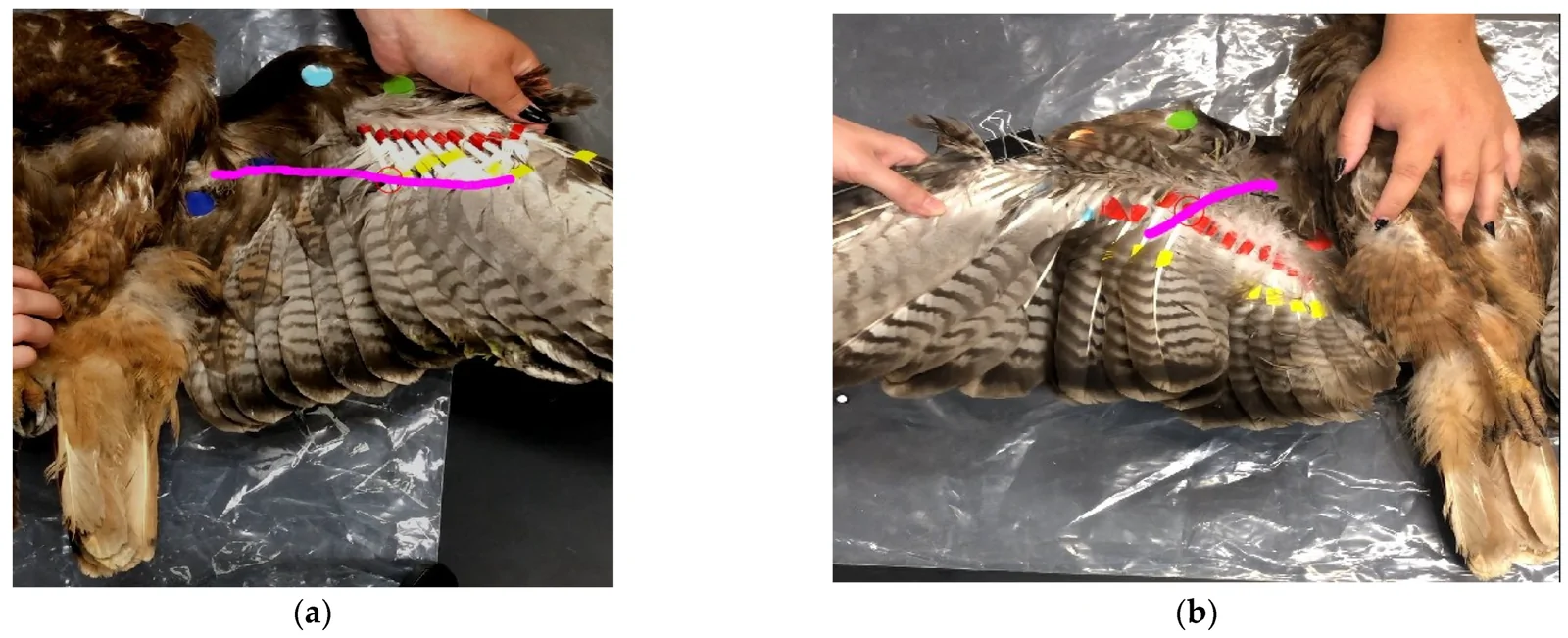
Motion capture on color-coded points on the (**a**) primaries and (**b**) secondaries.

Motion capture was performed on both the wing and tail to track feather motion during expansion and tucking. These measurements were used to help determine appropriate rubber band sizes and stiffnesses for the fabricated wing and tail mechanisms. For each feather, two solid-colored stickers were applied as tracking markers. One marker was wrapped around the feather shaft near its attachment point to the wing or tail, whereas a second marker was placed farther along the shaft, as shown in Figure 5a,b. These two points defined a vector along each feather shaft. In addition, three markers were placed at the finger, wrist, and elbow joints to track the motion of the wing skeleton.

A tripod-mounted camera was positioned above the hawk carcass to capture a top-down view of the wing and tail motion. During recording, the wing was manually and slowly extended to its maximum span before being fully tucked. The same procedure was repeated for the tail, which was manually expanded and tucked through its full range of motion. Multiple recordings were collected to ensure consistent marker visibility and adequate tracking coverage throughout the motion sequences.

**Figure 6:**
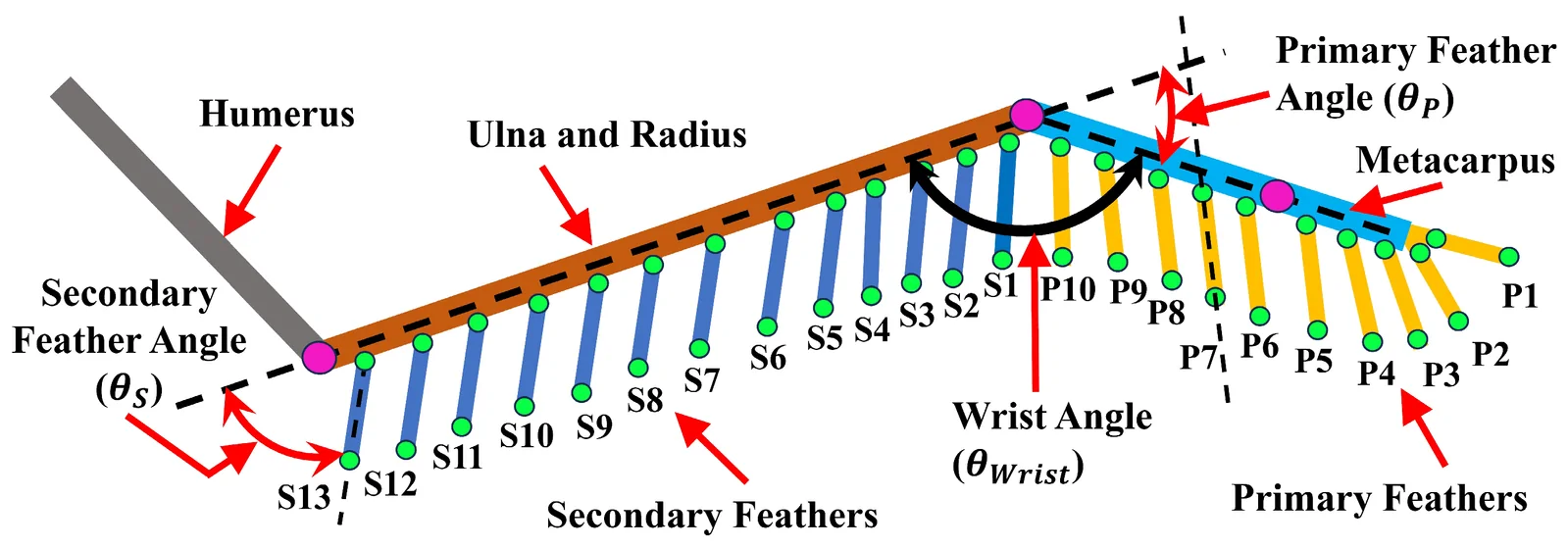
Primary and secondary feathers and wrist angles.

The recorded videos were processed in MATLAB using the DLTdv (version 8) digitizing tool. The tracking markers were automatically followed throughout the feather expansion and tucking motions, with minor manual corrections made whenever markers became temporarily obscured. The joint markers were used to form vectors along the ulna and metacarpus, allowing the wrist angle to be calculated, as shown in Figure 6. Similarly, vectors were formed along the shafts of the primary and secondary feathers. These feather vectors were then used to determine how each feather angle changes relative to the wrist angle as the wing was tucked and extended.

As demonstrated by PigeonBot I [14], the postpatagium, the connective tissue that interlaces through the flight feathers and extends toward the primary digits, can be modeled as a series of spring-like connections between feathers. These spring-like connections influence how rigid each feather moves during wing morphing. In the fabricated mechanism, orthodontic rubber bands were used to imitate this artificial postpatagium. Therefore, understanding each feather’s sensitivity to changes in wrist angle helped determine the relative stiffness needed for the rubber bands to reproduce similar feather positioning.

**Figure 7:**
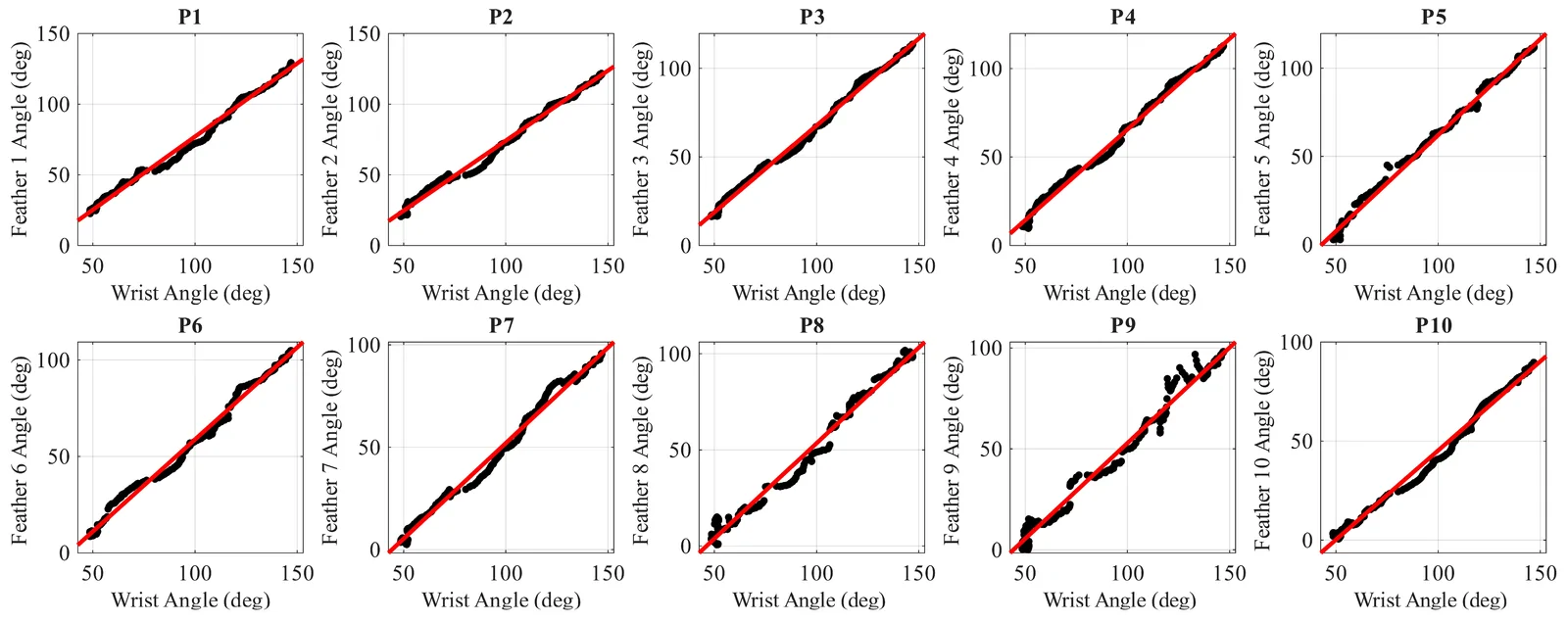
Sensitivity of primary feather angles to a change in the wrist angle (black dots: experimental measurements of feather angles; red line: linear curve fit).

**Figure 8:**
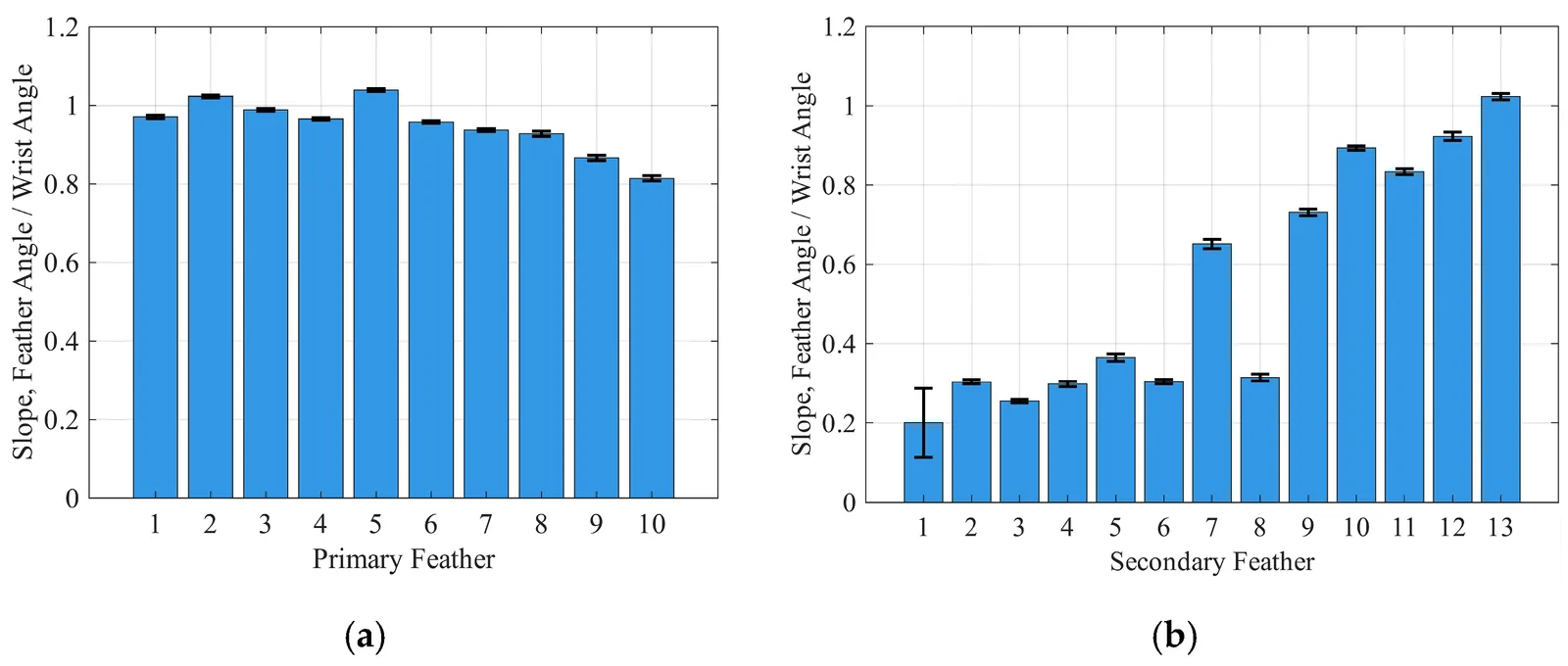
Sensitivity to wrist motion: (**a**) Primary feathers; (**b**) Secondary feathers.

For the kinematic analysis, the secondary feather angle, *θ_S_*, was defined as the angle between the ulna vector and the secondary feather shaft. The primary feather angle, *θ_P_*, was similarly defined as the angle between the ulna vector and the primary feather shaft. The wrist angle, *θ_wrist_*, was defined as the angle between the ulna and metacarpus vectors. The change in feather angle was plotted against the change in wrist angle during wing extension, as shown in Figure 7. A linear fit was then applied to each feather’s response, and the resulting slope was used as a measure of that feather’s sensitivity to wrist motion, as shown in Figure 8.

For the primary feathers, a feather that changes its angle slower relative to the ulna vector was interpreted as having lower rigidity relative to the metacarpus. In contrast, a higher sensitivity to wrist-angle change indicated greater rigidity. The feathers located closer to the distal metacarpus and primary digits showed the greatest sensitivity and therefore required the highest rigidity in the artificial mechanism. This result is reasonable because the finger joints themselves were not actively morphed during motion capture, meaning the distal primary feathers behaved more like stationary members. As the primaries approached the wrist joint, they moved more freely and rotated more closely with the wrist motion, indicating lower effective rigidity.

The secondary feathers showed a similar need for position-dependent stiffness, although their interpretation depended on their orientation relative to the ulna. Secondaries closer to the elbow joint tended to maintain their orientation more strongly as the wing morphed, indicating that they required stiffer artificial tissue connections to preserve their feather alignment. As the secondary feathers approached the wrist joint, they behaved more loosely and followed the wrist motion more closely. Therefore, these more distal secondary feathers were considered to require lower stiffness in the fabricated postpatagium mechanism.

### 2.4. Feather Extraction

**Figure 9:**
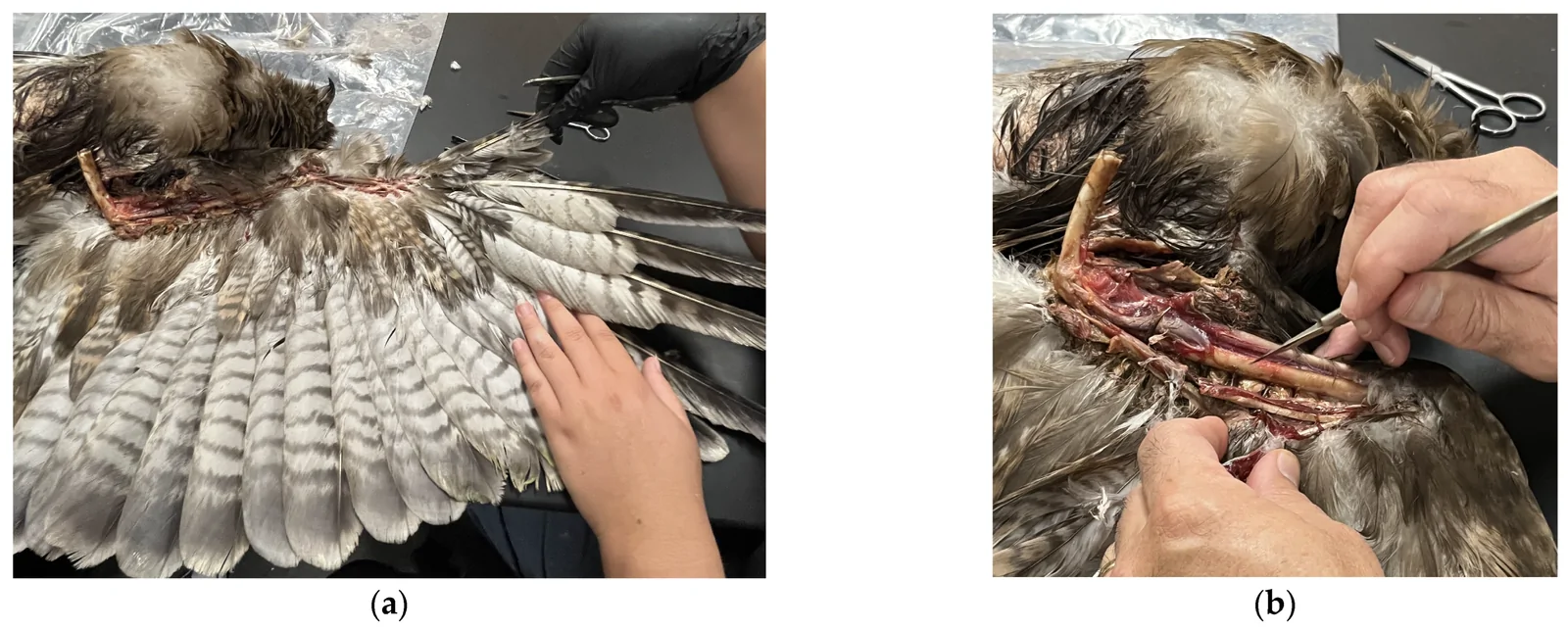
Feather extraction process: (**a**) Incision made along wing bones; (**b**) Exposed ulna and radius with postpatagial ligaments securing the feathers.

The wing feathers were removed from the hawk first. An incision was first made along the humerus and extended down the length of the ulna, radius, and metacarpus to expose the underlying bones, as shown in Figure 9a. Initially, it was believed that the feathers were attached directly to the bones through quill knobs, acting as pivot points for feather rotation, as described by Hieronymus et al. [28]. However, further dissection revealed that the secondary feathers were instead anchored to the skin by the postpatagial ligament (ligamentum postpatagiale), which in turn was attached directly to the quill knobs, as shown in Figure 9b. Each secondary and primary feather, excluding the digits, had a respective dorsal and ventral covert feather attached to it via the same postpatagial ligament, which rotated as a cohesive unit. The bases of all secondary and primary flight feather shafts ran along the side of the ulna and the metacarpus, respectively. The positioning of the feather shafts determined the placement of the feather holders in the wing mechanism (detailed in Section 2.6).

The primary flight feathers were removed by carefully cutting the postpatagial ligament, allowing the feathers to be extracted without structural damage while preserving the attached dorsal and ventral coverts. The skin over the metacarpus was desiccated, likely because of the limited fat content in this region and the resulting lack of moisture. As a result, the metacarpal skin detached completely from the wing, exposing the metacarpus and leaving skin only along the wrist joint. The secondary feathers were removed using the same general procedure, although less skin removal was required.

The tail feathers were removed by carefully cutting through the tissue near the base of the feather attachment region. Additional incisions were then made to expose more of the feather shafts until the feathers could be safely and easily extracted from the tail.

### 2.5. Carcass Dissection

**Figure 10:**
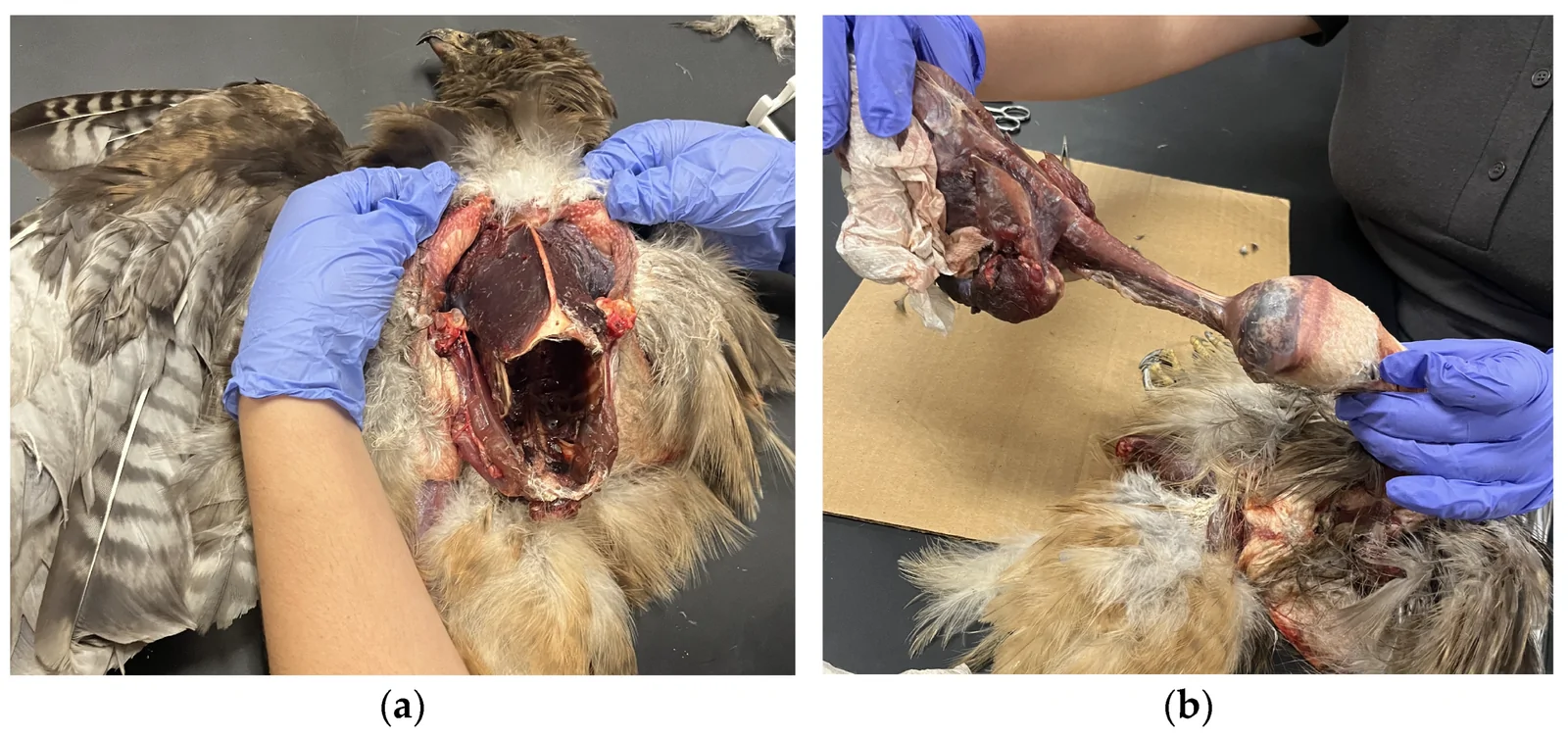
Skin separation process: (**a**) Separation of the skin from the tissue surrounding the sternum; (**b**) Removed torso with the attached neck separated from the skinned body while attached to the skull.

Once the feathers were removed, the internal organs and excess tissue were extracted to hollow out the body cavity and create space for the internal mechanisms. An initial incision was made along the belly near the hawk’s sternum, and the skin was carefully separated from the underlying tissue. The opening was then enlarged to allow for the removal of the internal organs. As the dissection progressed, additional cuts were made to gradually peel the skin away from the surrounding muscle and connective tissue, as shown in Figure 10a. The separation continued around the sternum so that the skin remained intact whereas the sternum and attached muscle tissue stayed together as a separate structure. This process continued until the skin was fully detached from the sternum and surrounding tissue, resulting in two separate components, as shown in Figure 10b. Excess tissue, including muscle surrounding the legs and clavicle region, was removed to further hollow out the body cavity.

Incisions were extended upward toward the neck and skull, exposing more of the internal tissue and musculature. As the dissection approached the head, the skin was carefully turned inside out to expose the connective tissue attaching it to the skull and surrounding structures, allowing the skin to be separated without damage. Once the back of the skull was fully exposed, a section of the skull was removed with a saw to access and extract the brain and eyes. The neck was then severed from the skull, allowing the previously separated torso structure to be fully removed from the skin and used for further 3D scanning.

### 2.6. Wing Design and Fabrication

**Figure 11:**
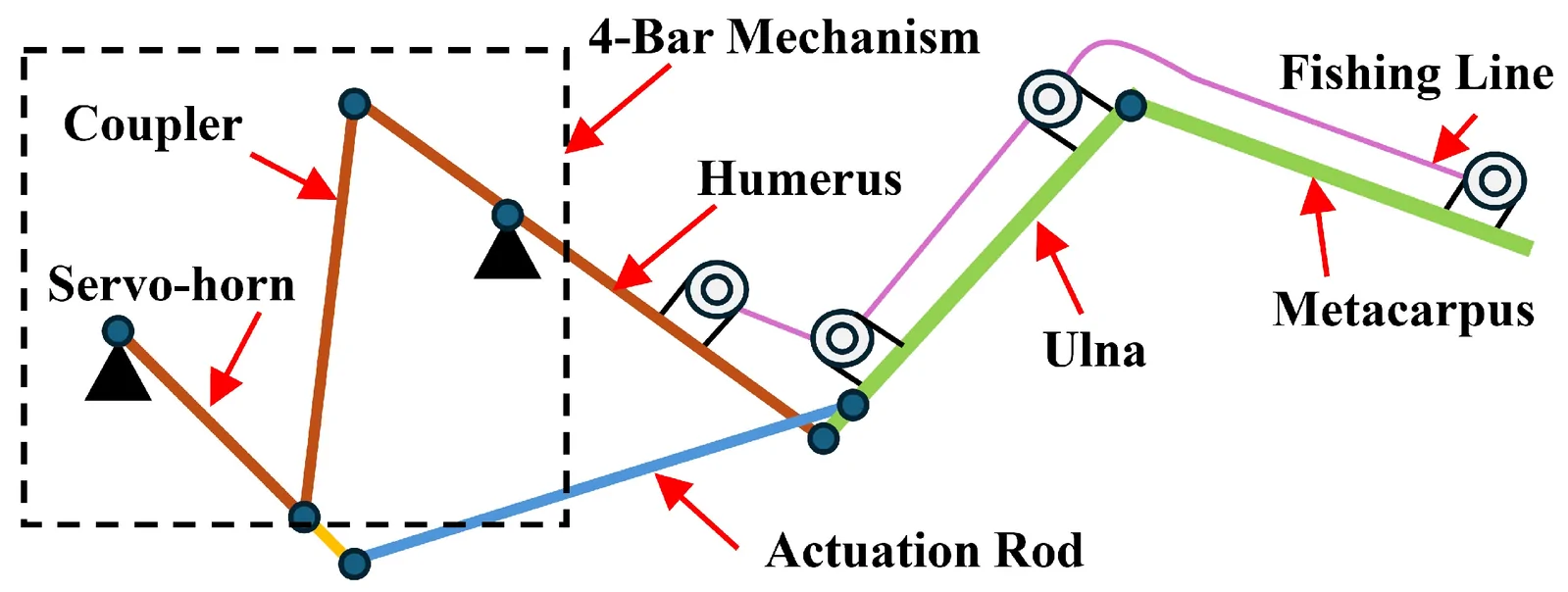
Kinematic diagram of wing mechanism (black triangle: fixed point to fuselage; blue dot: hinge; circle: channel where the fishing wire goes through).

**Figure 12:**
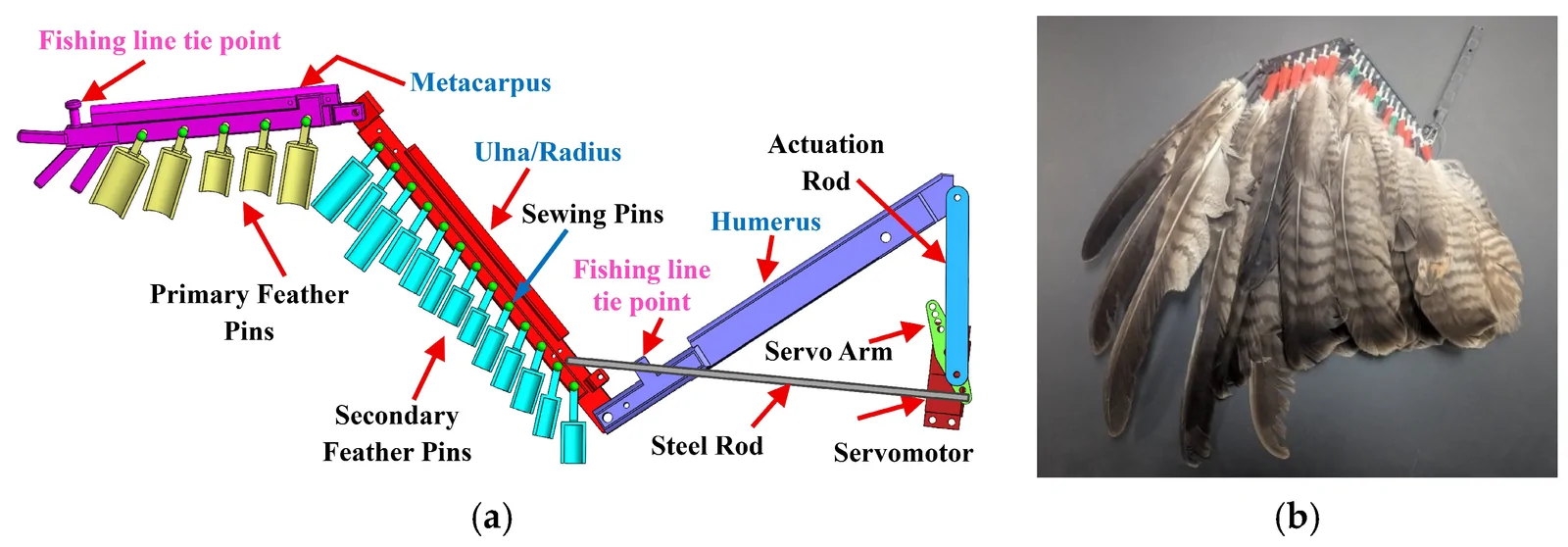
Wing assembly: (**a**) CAD model in SOLIDWORKS; (**b**) fabricated example.

A kinematic diagram of the wing morphing mechanism was first developed to aid in sizing the different links to achieve the required deformation. The diagram is shown in Figure 11. The detailed design of the wing morphing mechanism is shown in Figure 12a. It features one degree of freedom (DOF), enabling wing morphing to achieve tucked and extended configurations, with one MKS HV6150 servomotor per wing. It is composed of a 3D-printed polylactic acid (PLA) humerus, ulna, and metacarpus, created using a digitization tool, InkScape and online resource [29] to CAD the bones on SOLIDWORKS. This excludes the radius because an additional bone in the mechanism would not fit inside the skin during preliminary fitting checks. Combining the ulna and radius bones to one bone was carried out before in a previous morphing drone, raptor-inspired feathered drone [30]. A braided fishing line runs along the upper surfaces of the humerus, ulna, and metacarpus, passing through designed channels and looping around short steel rods positioned along the bones, acting as both a radius and a bicep muscle, aiding in wing morphing. Three-dimensionally printed feather pins are positioned along the dorsal side of the wing with sewing pins and earring backings keeping them in place. All flight feathers and their respective coverts are connected to the feather pins via glue and heat shrink, except for the five digits, which are connected in a similar way directly to the metacarpus with no ability to rotate. Flight feathers are interconnected via orthodontic rubber bands, which act as the postpatagium.

All joints on the wing mechanism are revolute for simplification and ease of manufacturing. The wrist joint, however, contains a 20° twisted housing on both the metacarpus and ulna in opposing directions. This joint connection between the ulna and the metacarpus was proposed by Tang et al. [27] to allow for the tucking of the metacarpus and primaries beneath the mid-wing.

The wing servomotor is housed within the fuselage, whose servo arm connects to two actuation rods, one of which connects to the outer end of the humerus, 3D-printed out of PLA, forming a 4-bar mechanism that actuates the humerus. The other actuating rod is a 2 mm steel rod, which is inserted into the most distal position on the servo arm and to the proximal point on the ulna. As the servo arm rotates (counterclockwise for the left wing), the humerus extends until the four-bar mechanism reaches its dead-center position. Along this rotation of the servo arm, the steel rod extends the ulna. Because the fishing line is tied to a distal point on the metacarpus and to the humerus, as the ulna extends, the fishing line lengthens and therefore pulls on the metacarpus. This, in turn, allows for the full extension of the wing. To return the wing to a tucked configuration, the compressive force of the orthodontic rubber bands pulls the metacarpus down as the ulna is retracted by the steel rod. The assembled left wing is shown in Figure 12b.

All components were 3D-printed to simplify the design process, enabling more complex shapes and tighter tolerances, since the wing mechanism had to fit inside the drying skin. The use of fishing line allowed for an additional link to be added to the mechanism at minimal cost to consume additional space in the skin, enabling the extension of the metacarpus without the need for a radius.

### 2.7. Tail Design and Fabrication

**Figure 13:**
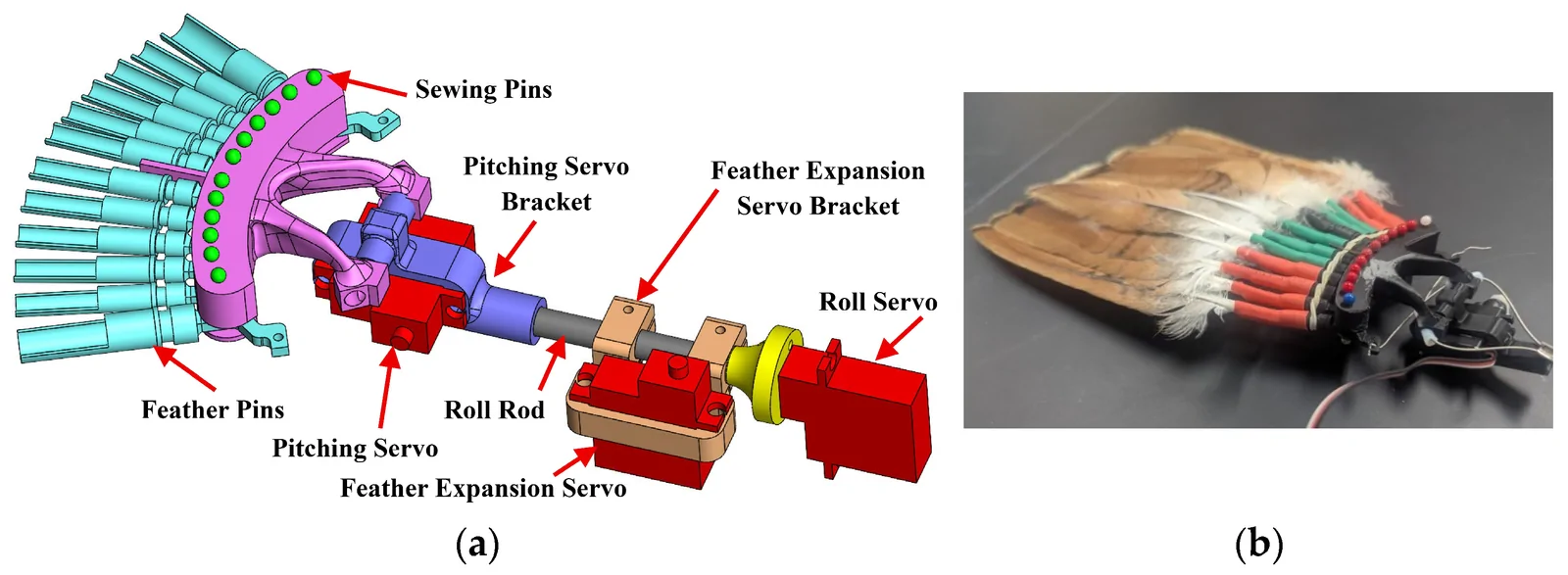
Tail assembly: (**a**) CAD model in SOLIDWORKS; (**b**) fabricated example.

The tail mechanism has three DOFs that include feather expansion, roll, and pitch, all actuated by three different DS-843MG servomotors, as shown in Figure 13a. The tail mechanism has a feather bracket that is connected to the pitching servo bracket, which in turn is assembled to the roll rod. The feather bracket supports twelve feathers, each of which is superglued and heat-shrunk onto a PLA feather pin. The pins are attached to the PLA feather bracket using sewing pins and earring backings, which both secures the feathers and allows them to move laterally from side to side. The feather bracket design is based on dimensions measured from the RTH, and Autodesk Fusion’s generative design (GD) tool was used to optimize the bracket geometry while minimizing material usage. During the GD process, the attachment points connecting the feather bracket to the roll rod were defined as fixed constraints, while loads were applied at the feather pin attachment locations and the pitch pivot point. Regions where the pins protruded were designated as obstacle geometry to prevent material generation in those areas. Orthodontic rubber bands are used to mimic elastic tendons by linking adjacent feathers together, allowing the feathers to either expand or tuck when the outer feather was pulled. Guitar string was attached to the outer feather pins and routed through PTFE (Polytetrafluoroethylene) tubing integrated into the feather bracket before being connected to the feather expansion servo. This mechanism enabled three distinct feather configurations: fully extended at 120°, mid-tuck, and fully tucked.

The feather bracket is connected to the PLA pitching servo bracket through a brass rod, which serves as the pivot point for pitching motion. A servomotor housed within the bracket actuates a pushrod connected to the feather bracket, enabling controlled upward and downward pitching movement. The roll rod is a 6 mm carbon fiber rod that was bonded to the pitching servo bracket, whereas the rolling servo was mounted to the opposite end using M2 bolts. The rod passes through the fuselage and supports the PLA feather expansion servo bracket along its length. This expansion bracket features a C-clamp mechanism that allows it to slide along the roll rod for positioning and then be tightened securely in place. Figure 13a shows the fabricated tail.

### 2.8. Fuselage and Neck Design and Fabrication

**Figure 14:**
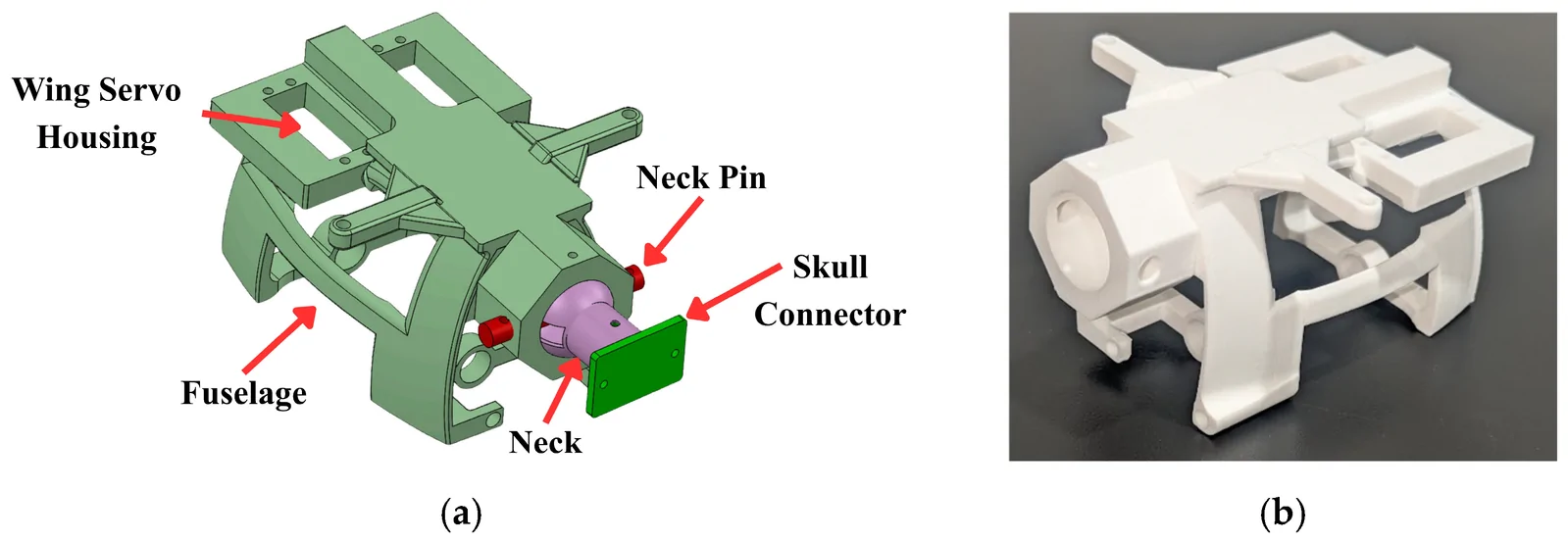
Fuselage structure: (**a**) CAD model in SOLIDWORKS with neck assembly; (**b**) 3D-printed example.

The fuselage structure, shown in Figure 14a, serves as the central structural component of the bird, connecting the wings, neck, and tail assemblies. It was designed using a 3D scan of the hawk’s interior skeleton as a reference geometry (as mentioned in Section 2.2). The leg bones and tail bone (pygostyle) were removed from the interior skeleton before the scan because the focus was integrating the wing, tail, and neck mechanisms. The resulting 3D scan was imported into SOLIDWORKS, where the fuselage geometry was modeled around the overall shape of the interior skeleton while maintaining its primary dimensions and incorporating a hollow internal structure. The bottom portion of the fuselage was sliced to account for skin shrinkage after drying and thus enabled the skin to be wrapped around the fuselage. Additional mounting features were integrated into the fuselage structure to support the tail roll rod, the two wing servos, and the neck structure. The 3D-printed fuselage structure is made of PLA and is shown in Figure 14b. Stretchy fabric was sewn onto the hawk’s skin at various points along the skin where Velcro strips were placed to cover the fuselage with cutouts for the connection of the wing mechanisms. Adding stretchy fabric to the bird’s skin was intended to minimize direct contact between the fuselage and the skin, preventing any tears in the skin as it wrapped around the fuselage.

**Figure 15:**
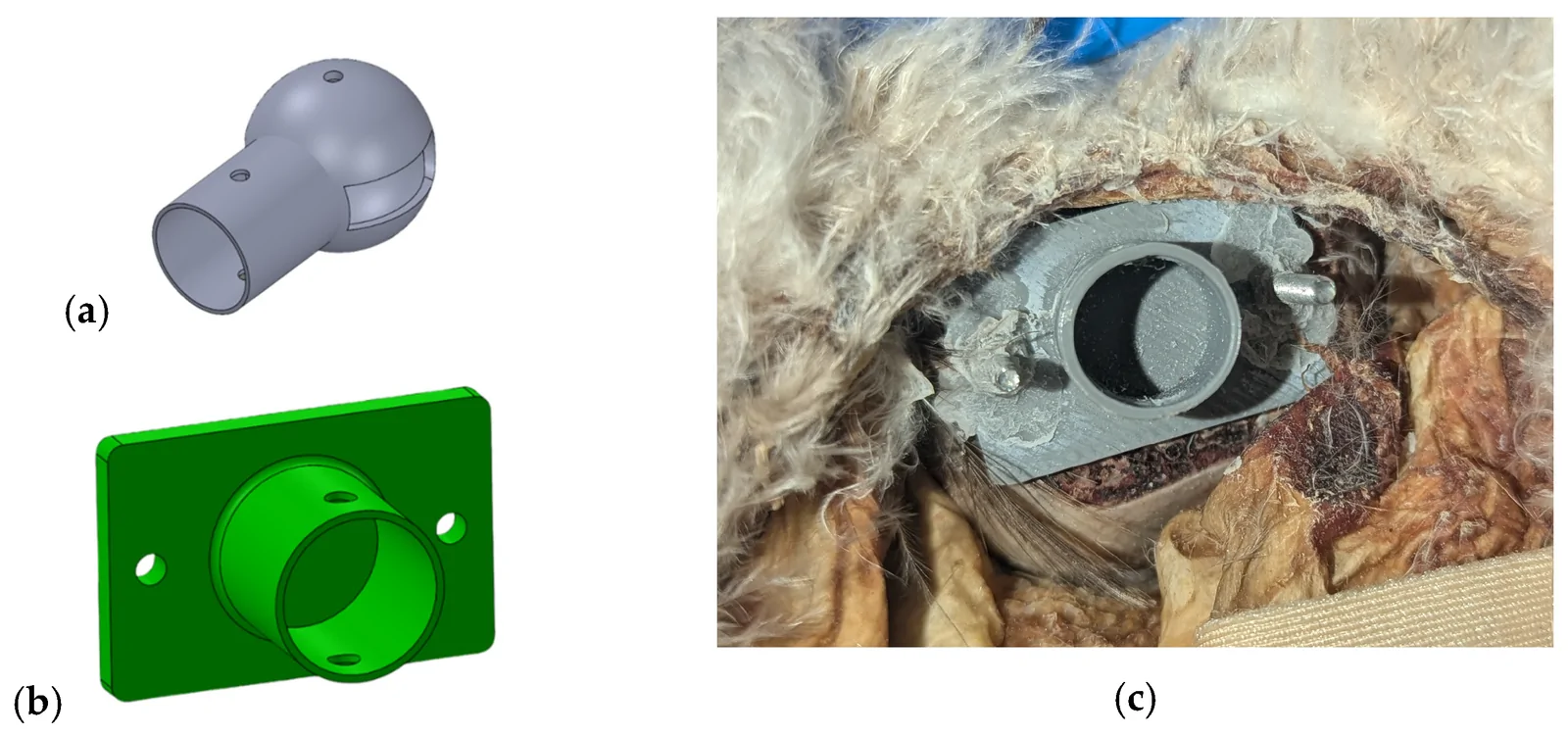
(**a**) Neck structure, (**b**) skull connector, and (**c**) skull connector attached to the RTH’s skull.

The neck assembly consists of a PLA cylinder designed to mimic the neck of the bird with a ball joint at one end, as shown in Figure 15a, that connects to the fuselage, as depicted in Figure 14a. The ball joint design allows the neck to take any possible orientation needed in flight studies. The connection point, where the neck connects to the fuselage, is an octagonal part with a space that fits the sphere of the neck ball joint. A PLA pin is inserted through the ball joint to secure the connection while restricting motion to the lateral direction. The opposite end of the rod is attached to the hawk’s skull using a PLA skull connector, as shown in Figure 15b, via M2 screws and nuts. This skull connector assembled to the prototype’s RTH skull is shown in Figure 15c. Additional M2 screws and nuts can be placed to hold the neck in a fixed position when neck movement is not desired. The neck orientation is adjusted manually without servomotors to keep weight to a minimum.

### 2.9. Assembling the RTH Taxidermy

**Figure 16:**
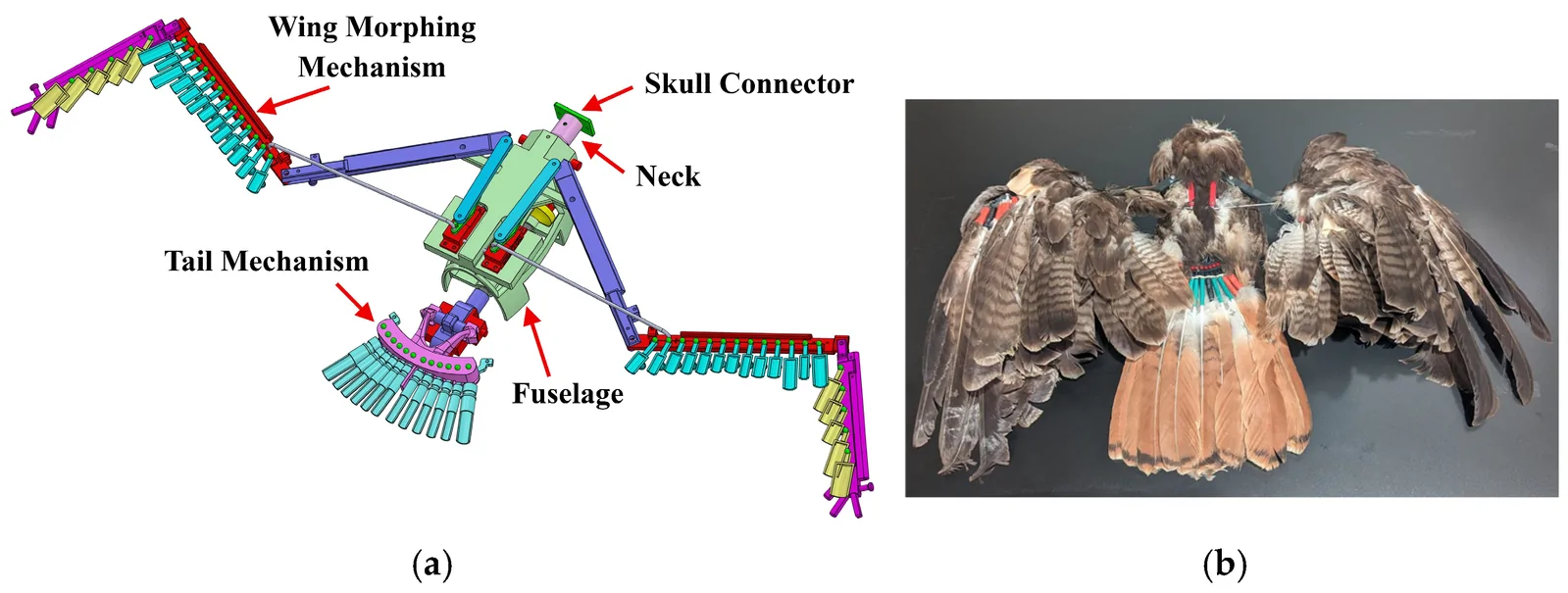
Full RTH assembly: (**a**) CAD model in SOLIDWORKS; (**b**) fabricated model.

The full morphing taxidermy model assembly, shown in Figure 16a, consists of the morphing wings, tail mechanism, and fuselage, with neck and skull connectors. All components were compared to the full 3D scan of the cadaver to confirm conforming sizing.

To attach the skin to the wings, swimwear fabric was sewn onto the skin to cover any gaps caused by desiccation from repeated cycles of use and the removal of all fat and tissue. The fabric allowed the skin to be wrapped around the ulna and the wrist joint. Holes designed near the elbow and wrist joints of the ulna enabled sewing pins and earring backings to be used to secure the skin to the wing mechanism. The loose coverts on the skin were secured to the wing mechanism by carefully tying them to the metacarpus and ulna with a braided fishing line.

The fuselage contains a Velcro strip on its bottom that is attached to the swimwear fabric by another piece of Velcro. The fabric is sewn onto the bird’s skin, encompassing the fuselage by one large piece of fabric where the two ends are attached together by sewn-on Velcro strips. The fabric aids in shielding the skin from tearing as the morphing taxidermy actuates its mechanisms. The wing and tail mechanisms are attached with the tail feather area exposed and its pitching bracket and rolling rod covered under the covert tail feathers and inside the fuselage. Figure 16b shows the assembly with the fuselage skin.

### 2.10. Avionics

**Figure 17:**
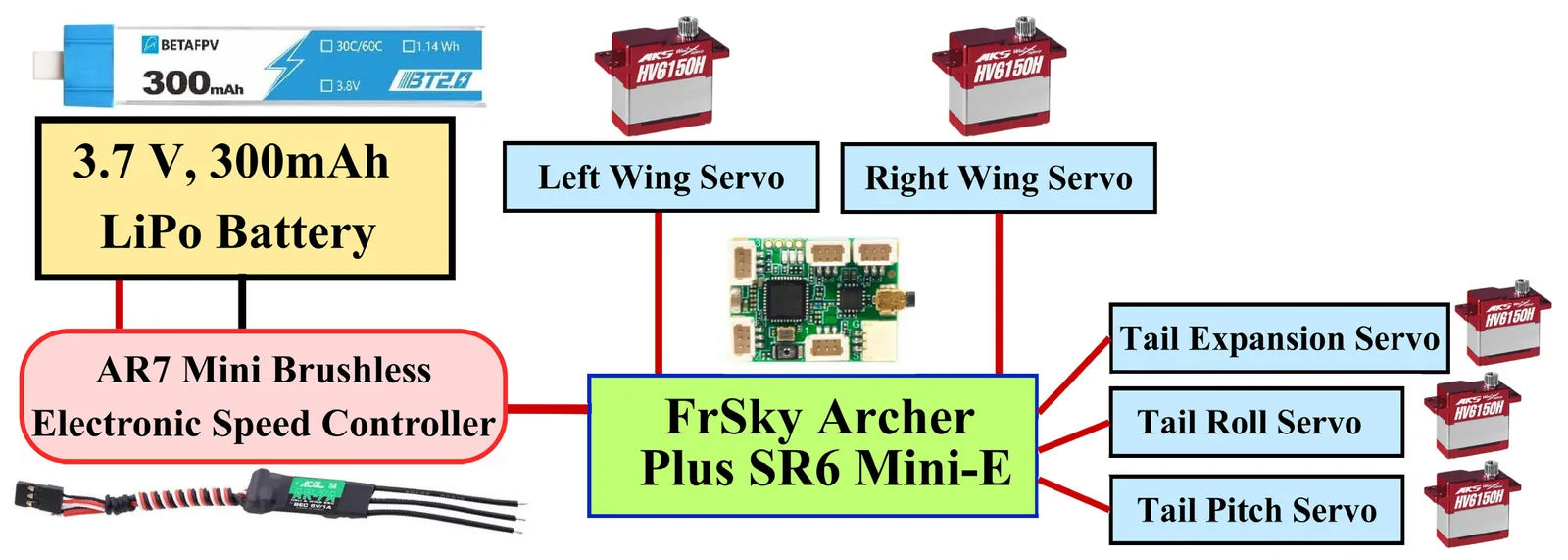
Avionics diagram.

Although this taxidermy model is not designed for flight, an avionics system similar to what can be found on a drone was selected for actuation and possible future addition of a propulsion system to enable the taxidermy to fly as a drone. A FrSky Archer Plus SR6 Mini-E 6 channel receiver is used for telemetry connection with a Taranis Qx7 radio transmitter. The receiver is powered by a BETAFPV, 3.7 V, 300 mAh lithium polymer battery via an AR7 mini brushless ESC that supplies 4.8 V to the actuation system. Figure 17 shows the avionics diagram.

## 3. Results

### 3.1. Rubber Band Stiffness Characterization

The stiffnesses of the rubber bands used in the wing and tail mechanisms were experimentally characterized to aid in selecting the rubber bands between each two neighboring feathers in the prototype to ensure the proper spreading of the feathers upon expansion without gaps or overlaps. Four types of Orthodontic rubber bands were considered, namely Chipmunk 3.5 oz and 6.5 oz and Rabbit 3.5 oz and 6.5 oz. The Chipmunk rubber bands had a nominal diameter of 3.2 mm, while the slightly larger Rabbit rubber bands had a nominal diameter of 4.8 mm. The 3.5 oz and 6.5 oz designations represent the manufacturer specified force ratings of the orthodontic elastics.

**Figure 18:**
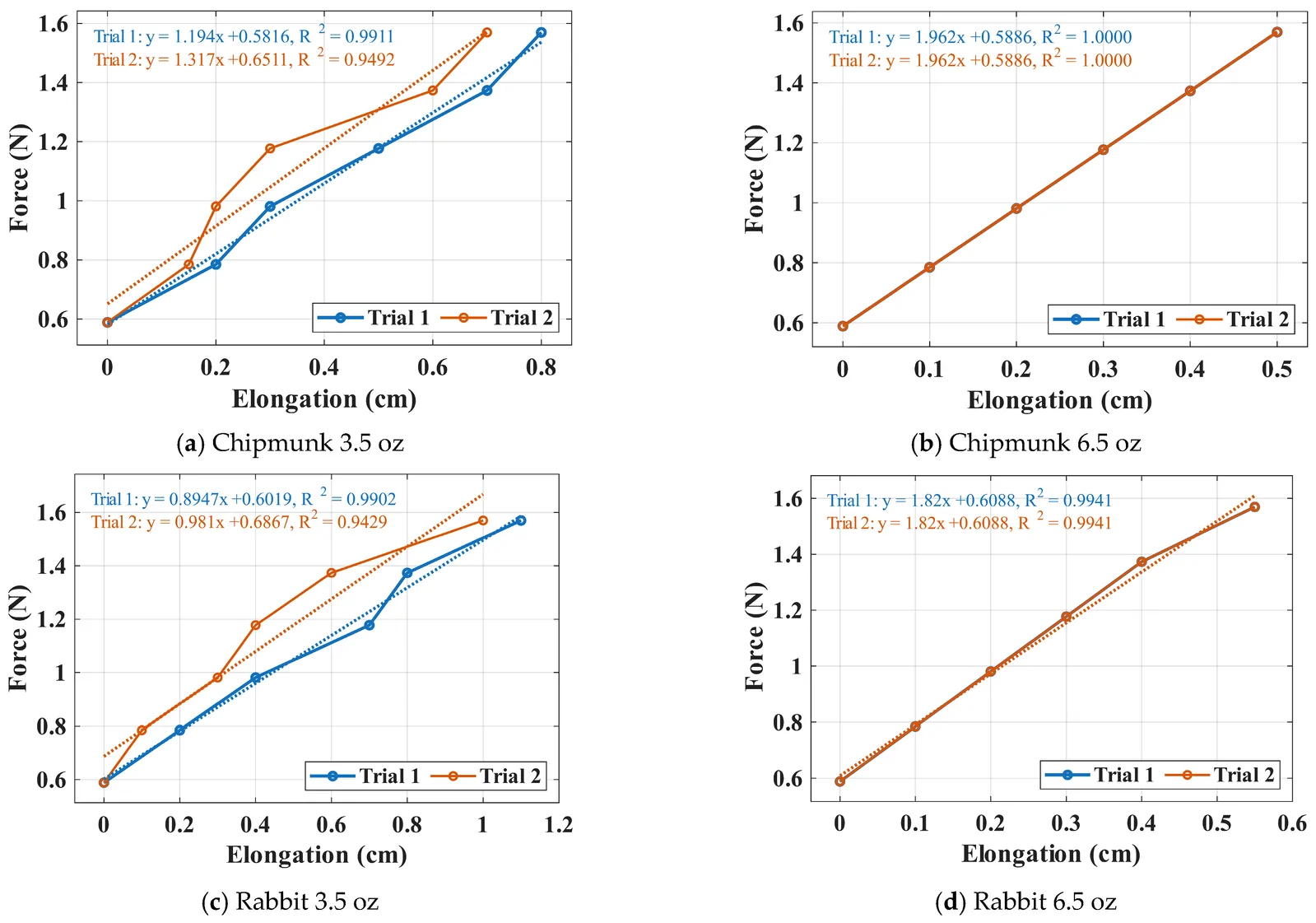
Force vs. Elongation graphs: (**a**) Chipmunk 3.5 oz, (**b**) Chipmunk 6.5 oz, (**c**) Rabbit 3.5 oz, and (**d**) Rabbit 6.5 oz.

The stiffness of each rubber band was determined by suspending a series of known weights from the rubber band and measuring the resulting displacement, as shown in Figure 18. For simplification, the rubber bands were initially assumed to exhibit linear elastic behavior in accordance with Hooke’s law. Linear regression curves were obtained, from which the stiffness was calculated for each rubber band, as reported in Table 2.

The 6.5 oz Chipmunk rubber band exhibited the greatest measured stiffness, consistent with its smaller diameter and higher force rating. The 6.5 oz Rabbit rubber band had the second highest stiffness, followed by the 3.5 oz Chipmunk and the 3.5 oz Rabbit rubber bands. The 3.5 oz rubber bands exhibited greater variation than the 6.5 oz rubber bands. This behavior is likely due to the elastic and nonlinear nature of rubber, which becomes more apparent as the applied load increases. For the loading range and trials used in this study, however, a linear approximation was considered acceptable because the deviations were small and the applied weights remained relatively low. The measured stiffness values were used to select suitable rubber bands for the wing and tail mechanisms, with stiffer rubber bands applied to feathers that showed lower sensitivity in accordance with the motion capture performed on the cadaver.

### 3.2. Mechanism Actuation Testing

**Figure 19:**
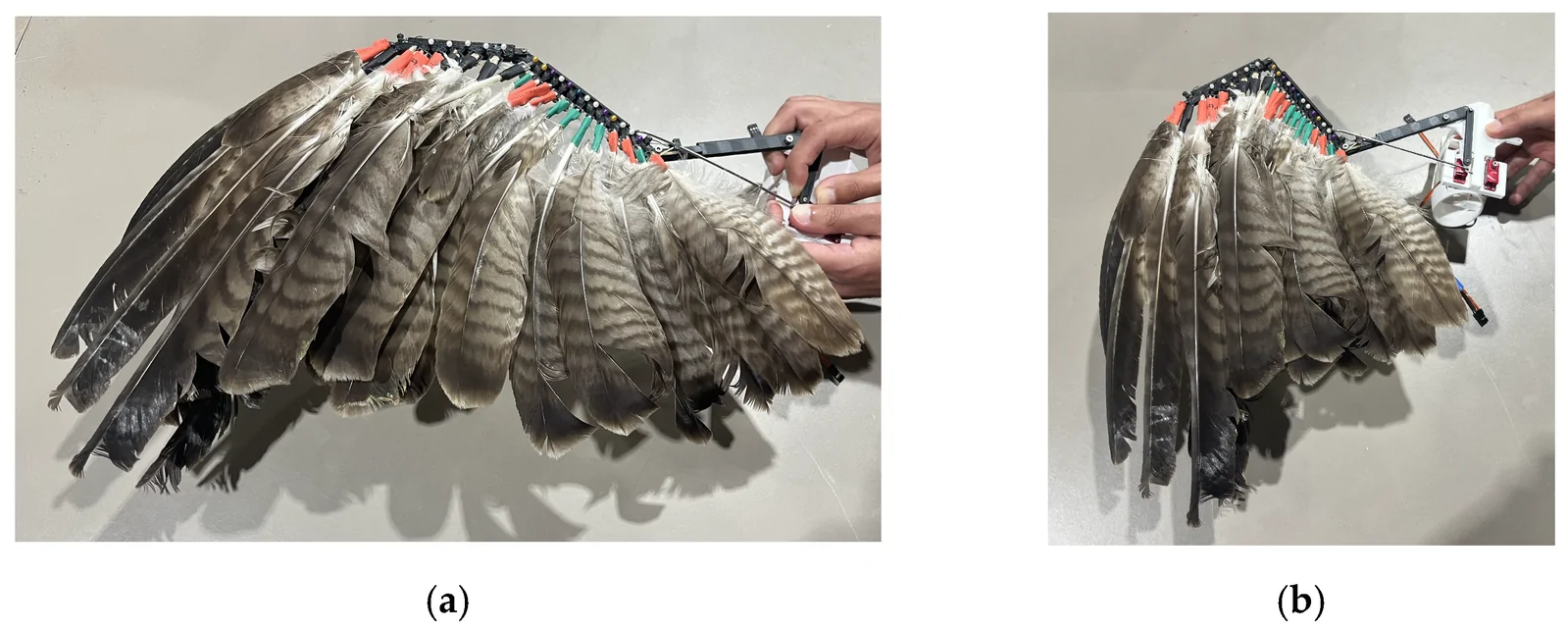
RTH morphing taxidermy wing: (**a**) extended and (**b**) tucked configurations.

Before integration into the RTH taxidermy, the wing mechanism was actuated to assess its functionality. The tests confirmed that the servo motors produced sufficient torque to overcome the pulling forces from both the orthodontic rubber bands and the fishing line. The wing achieved both extended and tucked configurations, as shown in Figure 19a,b, respectively, and reached the measured 145° wrist angle at full extension.

**Figure 20:**
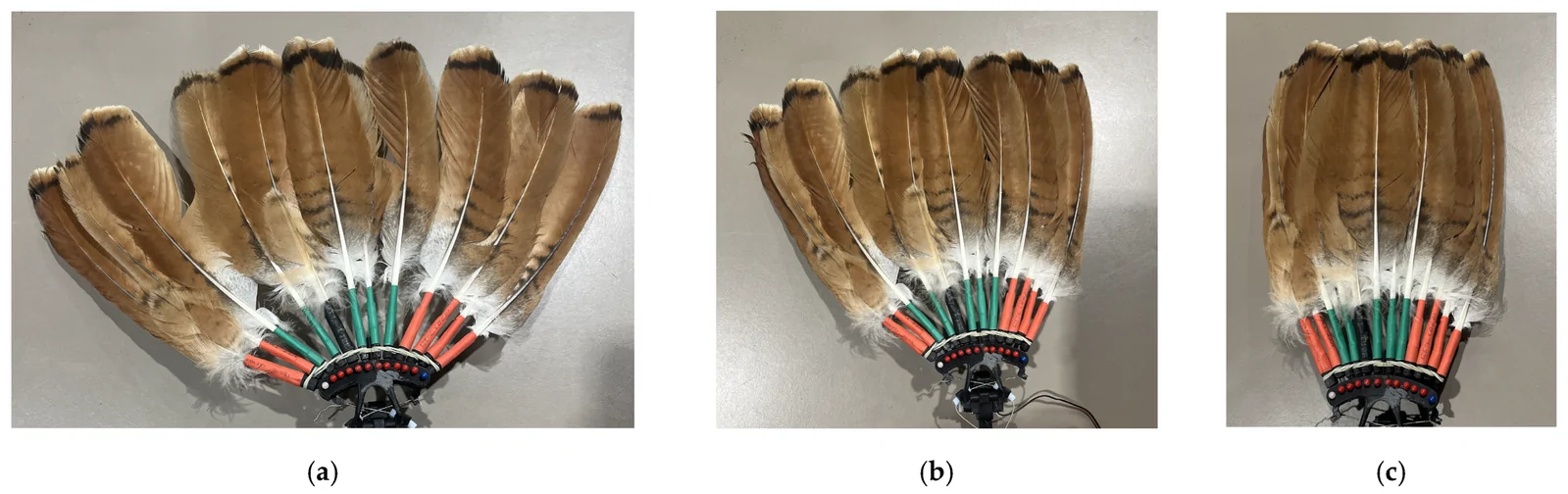
Tail actuation test (top view): (**a**) extended, (**b**) mid-tuck, and (**c**) fully tucked configurations.

**Figure 21:**
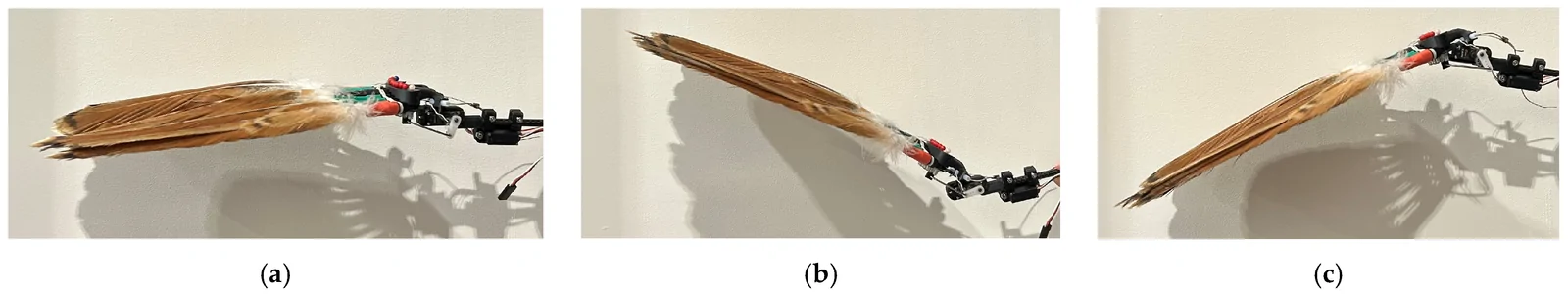
Tail actuation test (side view): (**a**) No pitch, (**b**) Pitched Up 30°, and (**c**) Pitched Down 30°.

The tail mechanism successfully achieved the three feather configurations: fully extended (Figure 20a), mid-tuck (Figure 20b), and fully tucked (Figure 20c). Additionally, the mechanism was capable of simultaneously performing rolling and pitching motions while reaching the targeted angular ranges and closely replicating the original dimensions and motion angles measured from the hawk. Figure 21a–c show the side of view of the tail with no pitch, 30° pitch up, and 30° pitch down, respectively.

### 3.3. Actuation Integrated in Taxidermy

**Figure 22:**
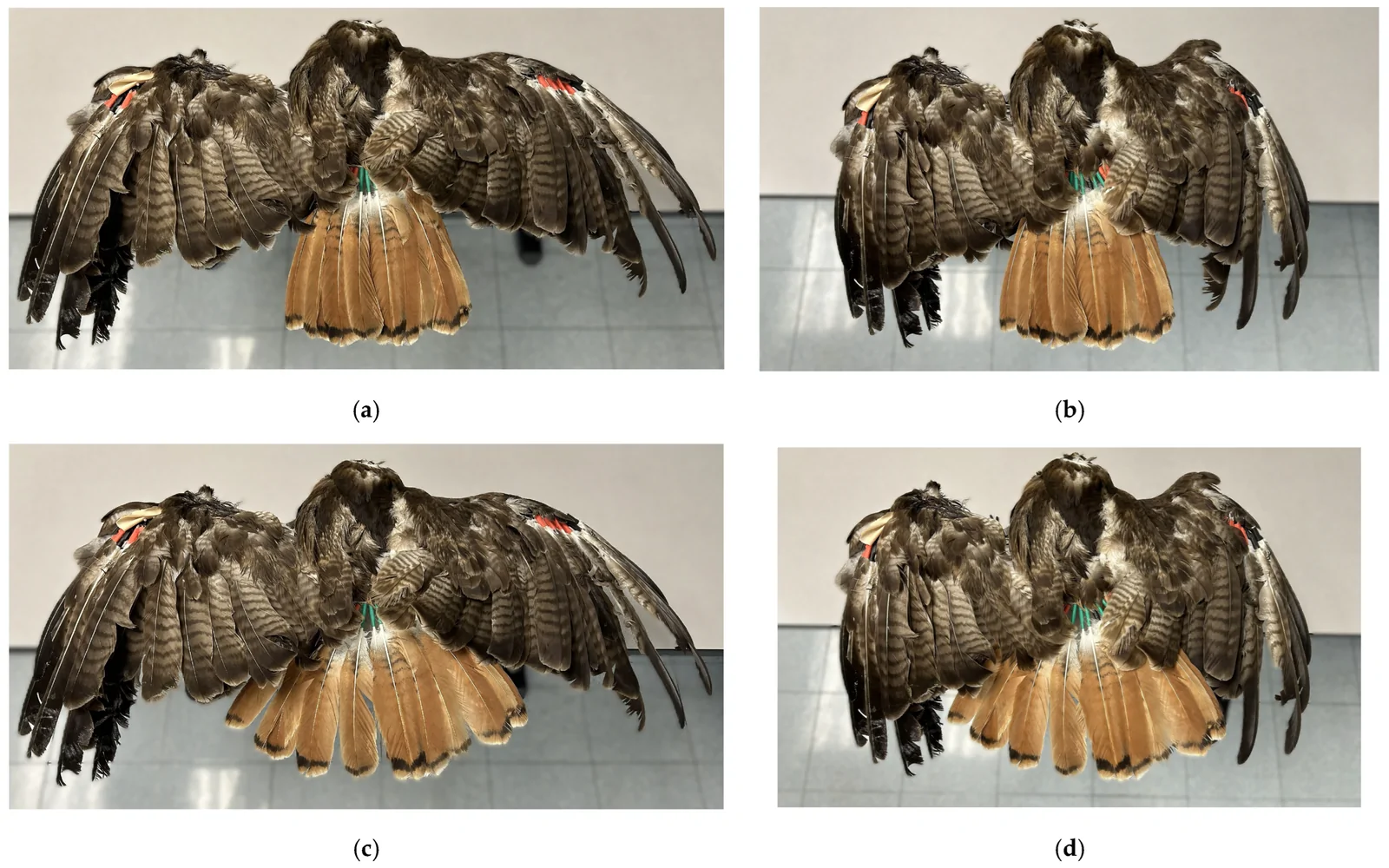
Full RTH morphing taxidermy prototype with (**a**) extended wings, tucked tail; (**b**) tucked wings, tucked tail; (**c**) extended wings, extended tail; and (**d**) tucked wings, extended tail.

The wing and tail mechanisms were successfully integrated inside the taxidermy and actuated. The wing and tail achieved their extended and tucked configurations as shown in Figure 22a–d. The feathers maintained their positions through repeated actuation tests. The skin remained securely attached and showed no signs of damage from the wing’s morphing or interference with the wing mechanism. The skin and taxidermy structure did not introduce any significant interference that prevented the tail from reaching its intended configurations. When integrated into the hawk carcass, the tail mechanism successfully achieved 30° upward and 30° downward pitching motion, 30° rolling motion to each side, and feather expansion from a fully tucked 60° configuration to the desired 120° expanded configuration.

Each degree of freedom was actuated multiple times, with the results showing that the mechanisms generally reached the intended angles. The achieved angle of each mechanism was recorded over 10 actuation cycles, with the mean angle and the standard deviation shown in Table 3. Both the left and right wings closely matched the target extended angle of 145°, achieving 145.95° and 145.60°, respectively, with small deviations. The tail pitching mechanism successfully reached the 30° target angle for both upward and downward motions, with relatively small deviations. The tail feather expansion reached 60° and 120° in the fully tucked and expanded positions, respectively. Throughout all 10 actuation cycles, no noticeable degradation in the performance of the actuators was noticed. Furthermore, the skin and suture remained intact throughout testing, with no indication of damage or loss of integrity.

Additionally, electric current measurements were taken for each degree of freedom over 10 actuation cycles, presented in Table 4. The current drawn by each servomotor was divided into three categories: idle, actuating, and end-position holding. In idle condition, the servomotors remained in neutral position while bearing minimal load. The actuating current represents the highest current demand while the servomotor was moving the mechanism between the idle and end positions. The end-position current represents the current required to hold the mechanism at an end position while resisting all forces produced by the mechanism.

The highest measured current was recorded for the left-wing servomotor while holding the mechanism at its end position. At the supplied voltage of 4.8 V, the measured current of 705.80 mA corresponds to a maximum power consumption of 3.39 W. This represents the worst-case operating condition and is not representative of continuous power demands, as the average power consumption of each mechanism requires only 335.10 mA, yielding an average power consumption of 1.61 W during continuous actuation. The AR7 mini brushless ESC successfully operated all degrees of freedom during repeated actuation tests without observed power loss. Therefore, the measured current demand was considered acceptable for the current prototype. However, a future flight-capable design would require a more substantial power system. During flight, in addition to the actuation system, the battery would supply additional avionics and propulsion components such as a flight controller, receiver, and a brushless DC motor. Additionally, multiple servomotors are often actuated simultaneously during flight, further increasing the total current demand. In such a case, rather than a 1S 3.7 V LiPo battery and an ESC supplying 4.8 V, a flight-ready design would use a battery with a higher voltage supply such as a 3S 11.1 V LiPo coupled with a BEC to provide 5–6 V to the servomotors. The exact power system would heavily depend on the drone’s total weight and the chosen motor.

### 3.4. Actuation Consistency

Motion-capture testing was conducted on the taxidermy morphing mechanisms after they were integrated into the cadaver. Because the wings and tail have symmetrical designs and rubber banding, data was collected only from the left wing and six of the twelve tail feathers. This reduced the amount of testing required while still providing a representative assessment of the overall mechanism. The aim of motion capture of the artificial wings and tail was to assess if consistent actuation has been achieved, repeatedly producing similar feather sensitivities and, therefore, angular positions.

**Figure 23:**
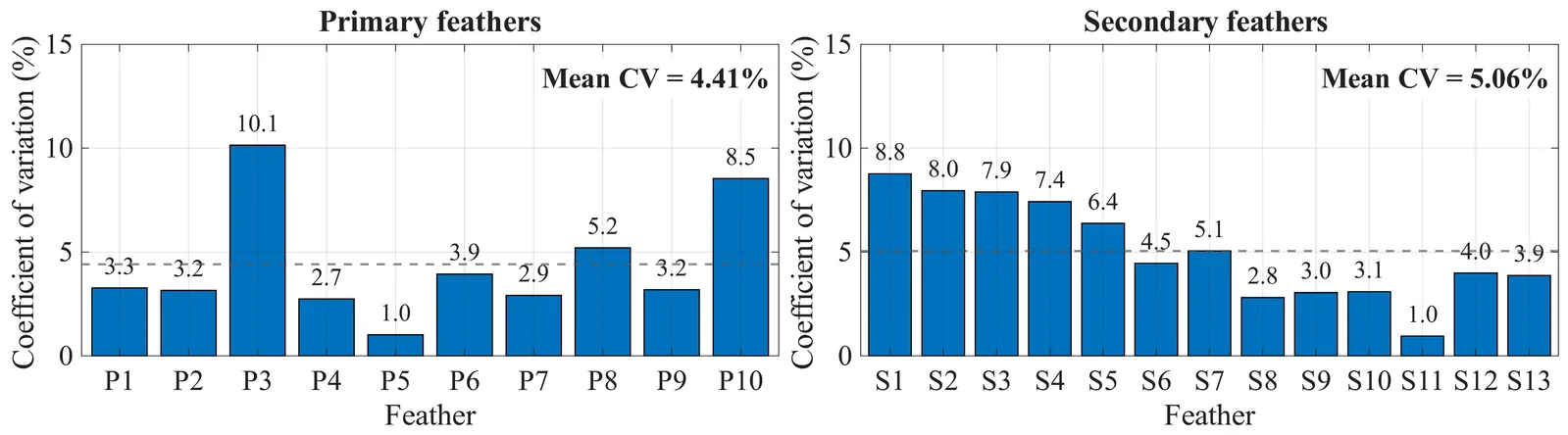
Coefficient of variation (CV) for the sensitivity of the wing feathers from motion capture (Horizontal dashed line represents the mean CV value).

**Figure 24:**
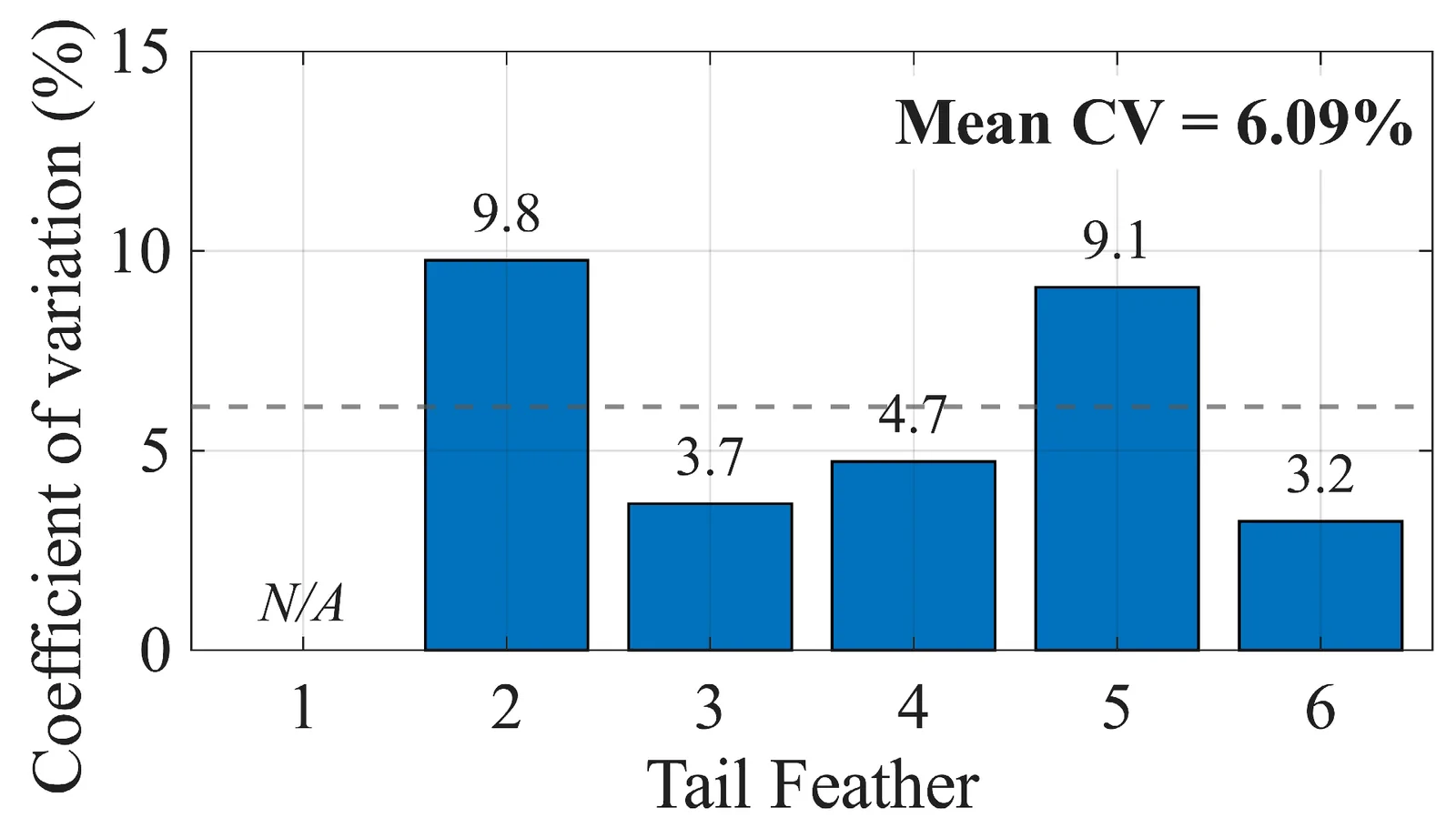
Coefficient of variation (CV) for the sensitivity of the tail feathers from motion capture (Horizontal dashed line represents the mean CV value).

The sensitivity of each feather was determined by measuring its change in position to the mechanism input, as discussed in 2.3. The mean sensitivity and standard deviation of each feather’s sensitivity were then calculated across five trials. The average sensitivity represented the typical response of each feather, while the standard deviation quantified the variation between repeated activations. As a measure of repeatability, the coefficient of variation (CV) for each feather’s sensitivity was calculated in both the wing and tail, as is shown in Figure 23 and Figure 24, respectively. The average CV for the primary and secondary feathers was 4.41% and 5.06%, respectively, while the tail showed an average CV of 6.09%. These results demonstrate that the feathers repeatedly spread approximately to the same positions and exhibited comparable sensitivity values, supporting the repeatability and reliability of the taxidermy’s morphing mechanisms.

## 4. Discussion

The morphing taxidermy of a Red-Tailed Hawk is unique in comparison to previous morphing wing models and drones. The uniqueness of the morphing taxidermy comes by utilizing the natural materials of the hawk. The natural feathers and skin served as the covering of the mechanisms rather than synthetic materials, such as composites, whose properties are difficult to adjust to match the natural parts. Utilizing the hawk’s natural body, fewer parts and materials were needed, minimizing the risk of causing the morphing taxidermy to be heavy. The natural flight feather of the hawk is difficult to replicate exactly with composites or balsa wood. Maintaining the exact feather with its stiffness saves time, effort, and cost in replicating the natural feather. The natural wings maintained the rest of the feathers intact after extraction of the primary and secondary feathers. The wings served as a guide to the placement of the wing mechanism.

With the limited types of available orthodontic rubber bands, selecting the rubber bands between any two feathers was a trial-and-error process. However, future studies will attempt to have a systematic approach to perform this tuning process, matching the measured sensitivities of the feather. Future work will include wind tunnel testing of the morphing taxidermy under various flight conditions and at different wing and tail configurations, measuring aerodynamic forces and the overall functionality of the morphing taxidermy. Additionally, future work will include adding more degrees of freedom, including wing pitch, tail sweep, and a controllable head.

The completed morphing taxidermy demonstrates that controlled wing and tail morphing functionality can be achieved while keeping its natural feathers and skin. The repeated actuation testing showed that the mechanisms could reliably imitate the intended wing and tail movement of a real red-tailed hawk with acceptable consistency between cycles. Although some asymmetry was observed between the wings in this proof-of-concept prototype, the overall results still support the idea of combining biological material with mechanical components.

Potential applications of a morphing taxidermy may include controlled wind tunnel testing and validation of computational models using biologically accurate wing and tail geometry. Furthermore, with future iterations including a propulsion system, this holds the potential for developing biomimetic UAVs, which would prove useful in cluttered aerial terrain where unconventional flight patterns, such as wing tuck and tail roll, provide an advantage. In addition, biomimetic UAVs may support ecological studies of avian creatures, allowing closer proximity during flight without scaring the wildlife away.

The current specimen was developed primarily as a proof-of-concept to demonstrate the proposed morphing mechanism rather than as a permanent device. All primary and secondary flight feathers and tail feathers were extracted from the skin and connected to their pins in the morphing mechanisms. Hence, these individual feathers are no longer directly attached to the biological skin. After removal of internal organs and adipose tissues, the skin was placed on the wing morphing mechanism and the fuselage to cover them. The completed morphing taxidermy prototype was kept in a plastic container box that gets bug-sprayed every week because the bird’s odor can attract ants and other bugs. Under laboratory storage conditions, the prototype remained mechanically compliant and functional for repeated morphing experiments for more than three months. No substantial degradation of the skin and feathers has been observed. Nevertheless, the long-term mechanical and biological stability levels of the specimen have not been systematically characterized, and the effective service life therefore remains to be quantified. Future work will investigate established taxidermy preservation techniques, including chemical tanning and tissue stabilization, to improve durability. In addition, artificial biomimetic skin materials that reproduce the mechanical characteristics of avian skin may provide a more robust and repeatable alternative for long-term operation and broader practical applications.

## 5. Summary and Conclusions

Despite previous research that incorporated bird taxidermies as fixed mounts or natural components of birds, such as feathers or a fixed taxidermy wing, in bioinspired drones, a morphing bird taxidermy has not been developed yet. The potential applications of a morphing taxidermy extend beyond aerodynamics. In ecological and behavioral studies, a dynamically adjustable specimen could provide more realistic visual stimuli for investigating predator–prey interactions, territorial behavior, mate choice, and social signaling. In flight research, it could serve as a bridge between rigid engineering models and live-bird experiments, enabling force measurements, flow visualization, stability analyses, and the validation of computational models under biologically realistic conditions. Such a platform could also facilitate the development of biomimetic morphing aircraft by providing experimentally validated insights into how birds use wings and tail morphing to achieve efficient and highly maneuverable flight.

Creating a bioinspired morphing taxidermy introduces uncommon engineering challenges from the integration of mechanical morphing mechanisms within an avian taxidermy. This paper proposes an approach to developing a morphing red-tailed hawk taxidermy that can be applied to similar sized birds. The proposed design with the selected small and lightweight structures enabled locating all electronic components within the small volume of the bird’s body. The developed prototype featured three morphing degrees of freedom in the tail and one independent degree of freedom in each wing, enabling many configurations for various aerodynamic and ecological studies. This achievement verifies the possibility of manufacturing biomimetic drones using both biological materials and mechanical components.

## Figures and Tables

**Table 1:** RTH carcass measurements.

Parameter	Value	Parameter	Value
Wingspan	114.3 cm.	Ulna Approx. Length	15.24 cm.
Head to Tail Length	50.8 cm.	Tail Length	24.13 cm.
Body Width	15.24 cm.	Tail Width (Tucked) along feathers	7.62 cm.
Weight	872 g	Tail Angle (Tucked)	0°
Humerus Approx. Length	11.43 cm.	Tail Width (Extended) along feathers	38.1 cm.
Radius Approx. Length	15.24 cm.	Tail Angle (Extended) along feathers	120°
Wrist Angle (Extended)	145°	Tail Root Width	~12.7 cm.
Wrist Angle (Tucked)	40°		

**Table 2:** Rubber band measured stiffness values.

Rubber Band	Trial 1 (N/cm)	Trial 2 (N/cm)	Mean (N/cm)	Standard Deviation
Chipmunk (3.5 oz)	1.19	1.32	1.26	0.092
Chipmunk (6.5 oz)	1.96	1.96	1.96	0.000
Rabbit (3.5 oz)	0.89	0.98	0.94	0.061
Rabbit (6.5 oz)	1.82	1.82	1.82	0.000

**Table 3:** Target and measured angles for each degree of freedom (10 actuation cycles).

DOF/Position	Target Angle	Measured Mean Angle	Standard Deviation
Right Wing Tucked	90°	90.23°	1.32°
Right Wing Extended	145°	145.60°	1.46°
Left Wing Tucked	90°	88.58°	2.61°
Left Wing Extended	145°	145.95°	1.60°
Tail Pitch Up	30°	30.24°	0.76°
Tail Pitch Down	30°	30.49°	2.76°
Tail Feather Fully Expanded	120°	120.43°	2.30°
Tail Feather Mid-Tucked	75°	75.02°	3.87°
Tail Feather Fully Tucked	60°	61.91°	2.31°

**Table 4:** Measured servo current in each degree of freedom (10 actuation cycles).

Servo	Servo/DOF	Idle Current	Actuating Current	End-Position Holding Current
MKS HV6150H	Left Wing	62.10 mA	335.10 mA	705.80 mA
	Right Wing	57.20 mA	352.20 mA	697.50 mA
DS-843MG	Tail Pitch	64.40 mA	261.70 mA	452.70 mA
	Tail Roll	38.80 mA	225.60 mA	516.10 mA
	Tail Expansion	32.20 mA	181.30 mA	292.30 mA

## Data Availability

The data supporting the findings of this study are available within the article and its Supplementary Materials.

## Supplementary Materials

The following supporting information can be downloaded at https://www.mdpi.com/article/10.3390/biomimetics11080559/s1. Video S1: Morphing RTH actuation tests.

## Appendix A

**Table A1:** The proposed morphing taxidermy vs. previous morphing drone models.

Morphing Model	Proposed Morphing Taxidermy	Ornithopter	PigeonBot I	LisHawk	MataGull
Inspired Bird	Red-Tailed Hawk	Pigeon and Pheasant	Pigeon	Northern Goshawk	Seagull
Objective	Develop a morphing taxidermy model using the carcass of a Red-Tailed Hawk.	Explore three types of camouflage drone designs: fixed-wing, quadcopter, and ornithopter for wildlife surveillance or military.	Develop a biohybrid morphing wing with real feathers to understand the wing morphing.	Develop a drone to show that adapting the wing and tail surfaces can improve agility, maneuverability, stability, flight speed range, and required power usage.	Create a bio-inspired, non-flapping, morphing drone resembling a seagull.
Number of DOFs	Wings: 1 Tail: 3	Wings: 1 Tail: N/A	Wings: 1 Tail: 2	Wings: 1 Tail: 2	Wings: 2 Tail: 3
Wing Sweep Range	90–145°	N/A; flapping.	0–60°	45–130°	0–70°
Tail Sweep, Pitch, Roll	Sweep: 0–120° Pitch: ±30° Roll: ±30°	N/A	N/A	Sweep: 10–60° Pitch: ±20° Roll: N/A	Sweep: 30–90° Pitch: +40°, −60° Roll: ±30°
Materials	Natural skin, natural feathers, PLA, guitar string.	Pigeon natural feathers, Pheasant natural tail feathers, Pheasant natural skin.	Natural feathers, balsa wood, Nylon, foamboard, elastic bands, cardstock.	Fiberglass, ribstop polyester fabric, EPP * foam, ABS ^†^, balsa wood, carbon shafts.	PLA, EPP foam skin, PCTPE ^‡^, balsa wood, carbon fiber, TPU ^§^, polystyrene.
Actuation Type	Wings: four-bar linkage. Tail: Pull design with music wire and carbon rod.	Wings: flapping actuation system. Tail: N/A.	Wings: elastic ligament. Tail: elevator and rudder design.	Wings: linkage, hinge joint, elastic tendons. Tail: Elevator and rudder by a universal joint and linkages.	Wings: hinge mechanism, FishBAC-inspired design. Tail: Compliant mechanism.
Actuation Torque	6.3 kg-cm	Not specified	0.8 kg-cm	3.8 kg-cm	4.8 kg-cm
Weight	375 g	Not specified	280 g	284 g	991 g

* Expanded Polypropylene; ^†^ Acrylonitrile Butadiene Styrene; ^‡^ Plasticized Copolyamide Thermoplastic Elastomer; ^§^ Thermoplastic Polyurethane.
